# RNF8 and SCML2 cooperate to regulate ubiquitination and H3K27 acetylation for escape gene activation on the sex chromosomes

**DOI:** 10.1371/journal.pgen.1007233

**Published:** 2018-02-20

**Authors:** Shannel R. Adams, So Maezawa, Kris G. Alavattam, Hironori Abe, Akihiko Sakashita, Megan Shroder, Tyler J. Broering, Julie Sroga Rios, Michael A. Thomas, Xinhua Lin, Carolyn M. Price, Artem Barski, Paul R. Andreassen, Satoshi H. Namekawa

**Affiliations:** 1 Division of Reproductive Sciences, Division of Developmental Biology, Perinatal Institute, Cincinnati Children’s Hospital Medical Center, Cincinnati, Ohio, United States of America; 2 Department of Pediatrics, University of Cincinnati College of Medicine, Cincinnati, Ohio, United States of America; 3 Department of Obstetrics and Gynecology, University of Cincinnati College of Medicine, Cincinnati, Ohio, United States of America; 4 State Key Laboratory of Genetic Engineering, Institute of Genetics, Collaborative Innovation Center of Genetics and Development, School of Life Sciences, Fudan University, Shanghai, China; 5 Department of Cancer Biology, University of Cincinnati College of Medicine, Cincinnati, Ohio, United States of America; 6 Division of Allergy and Immunology, Division of Human Genetics, Cincinnati Children’s Hospital Medical Center, Cincinnati, Ohio, United States of America; 7 Division of Experimental Hematology and Cancer Biology, Cincinnati Children’s Hospital Medical Center, Cincinnati, Ohio, United States of America; MRC Human Genetics Unit, UNITED KINGDOM

## Abstract

The sex chromosomes are enriched with germline genes that are activated during the late stages of spermatogenesis. Due to meiotic sex chromosome inactivation (MSCI), these sex chromosome-linked genes must escape silencing for activation in spermatids, thereby ensuring their functions for male reproduction. RNF8, a DNA damage response protein, and SCML2, a germline-specific Polycomb protein, are two major, known regulators of this process. Here, we show that RNF8 and SCML2 cooperate to regulate ubiquitination during meiosis, an early step to establish active histone modifications for subsequent gene activation. Double mutants of *Rnf8* and *Scml2* revealed that RNF8-dependent monoubiquitination of histone H2A at Lysine 119 (H2AK119ub) is deubiquitinated by SCML2, demonstrating interplay between RNF8 and SCML2 in ubiquitin regulation. Additionally, we identify distinct functions of RNF8 and SCML2 in the regulation of ubiquitination: SCML2 deubiquitinates RNF8-independent H2AK119ub but does not deubiquitinate RNF8-dependent polyubiquitination. RNF8-dependent polyubiquitination is required for the establishment of H3K27 acetylation, a marker of active enhancers, while persistent H2AK119ub inhibits establishment of H3K27 acetylation. Following the deposition of H3K27 acetylation, H3K4 dimethylation is established as an active mark on poised promoters. Together, we propose a model whereby regulation of ubiquitin leads to the organization of poised enhancers and promoters during meiosis, which induce subsequent gene activation from the otherwise silent sex chromosomes in postmeiotic spermatids.

## Author summary

To produce unimpaired sperm, precise activation of germline-specific genes is an essential step during the late stages of spermatogenesis. However, sex chromosomes carrying these genes become silenced in a chromosome-wide manner during meiosis in a process called meiotic sex chromosome inactivation. Sex chromosome inactivation is maintained from meiosis into postmeiotic spermatids. Thus, to ensure the function of sex chromosome-linked (sex-linked) genes required for male reproduction, these genes must escape silencing for activation in spermatids. Here, we unravel the epigenetic mechanisms that underlie the activation of sex-linked genes from otherwise inactive sex chromosomes in the male germline. We determine the mechanism by which two factors regulate gene activation: one is RNF8, a DNA damage response protein, and the other is SCML2, a germline-specific Polycomb protein. Our data suggest that, during meiosis, RNF8 and SCML2 cooperate to regulate ubiquitination, which establishes active epigenetic modifications on enhancers and promoters for subsequent gene activation; these memories are maintained through meiotic divisions to induce gene activation in spermatids. Importantly, this study uncovers novel epigenetic mechanisms that underlie specific gene activation in spermatids and illuminates potential causes of male infertility.

## Introduction

Worldwide, 15% of couples have difficulty conceiving a child. In situations of male infertility, approximately 90% of cases are the result of sperm abnormalities [1]. Male infertility is a complex condition with an estimated 15% of cases caused by genetic disorders. However, the etiology of male infertility remains unknown for 40% of cases, which are thus termed idiopathic [2]. To produce unimpaired sperm, precise regulation of germline-specific genes is essential during the late stages of spermatogenesis. These genes are preferentially encoded by the sex chromosomes and have specialized functions in reproduction [3]. Dysregulation leads to sperm abnormalities commonly related to male infertility [4–12]. In vitro fertilization (IVF) is a major form of treatment for infertility, but a high failure rate persists, stemming in part from sperm abnormalities [13]. Although the activation of sex-linked genes in late spermatogenesis is a critical step for sperm maturation, the mechanism that underlies this activation remains largely unknown.

**Fig 1:**
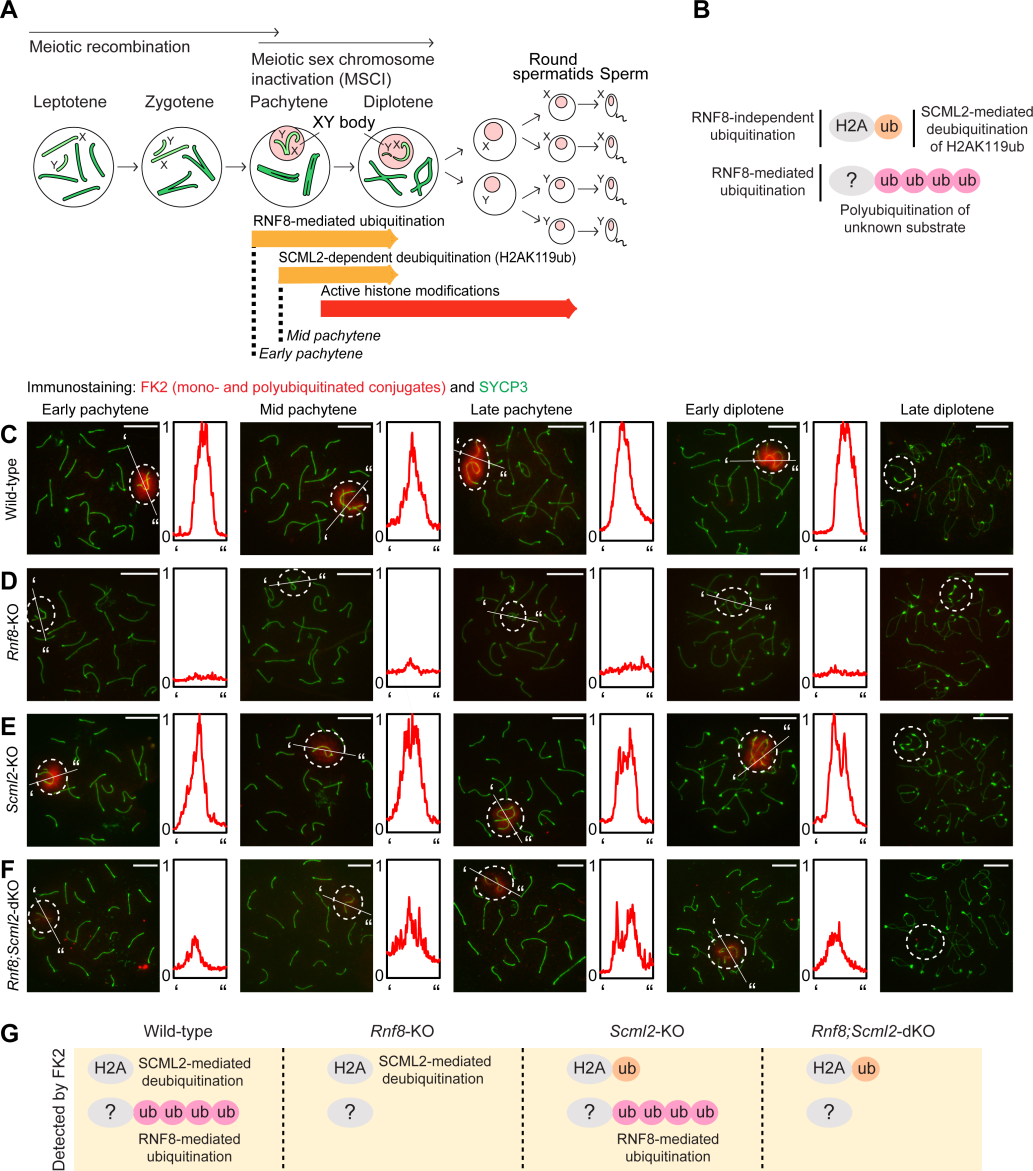
Distinct forms of ubiquitin regulation by RNF8 and SCML2: Immunostaining with FK2 antibody, which recognizes both mono- and polyubiquitinated conjugates. (**A**) Schematic of spermatogenesis. (**B**) Model of distinct forms of regulation of ubiquitination by RNF8 and SCML2. (**C-F**) Immunostaining of SYCP3 and FK2 on meiotic chromosome spreads. Dotted circles: sex chromosomes. Scale bar: 10 μm. Representative images are shown for at least 30 spermatocytes from each substage, from at least 3 independent mice per mouse model. The intensity of immunostaining is quantified by densitometry across the indicated path (‘ to”) and plotted in a relative intensity range of 0–1, which is normalized among the samples at the same stage. (**G**) Schematic of ubiquitin targets recognized by the FK2 antibody in each mouse model. A pink circle denotes RNF8-dependent ubiquitination and an orange circle denotes events mediated by a different E3 ubiquitin ligase.

Meiosis is the central event in germ cell development, followed by postmeiotic stages that form round spermatids and then mature sperm. During male meiosis, in response to the lack of synapsis, the X and Y sex chromosomes undergo forms of regulation distinct from synapsed autosomes, inactivated in a process known as meiotic sex chromosome inactivation (MSCI) (Fig 1A). MSCI is an essential event in germ cell development that involves almost complete chromosome-wide silencing [14–19], and this chromosome-wide silencing is maintained into postmeiotic spermatids following two rounds of meiotic division [19, 20]. However, a relatively large group of sex-linked male reproduction genes (~100 genes) escape from chromosome-wide silencing for activation in postmeiotic spermatids, ensuring their functions for male reproduction [19–22]. The mechanism by which genes escape from sex chromosome inactivation to become activated persists as an unsolved mystery.

DNA damage response (DDR) pathways are adapted to act on the meiotic sex chromosomes to initiate MSCI [23]. At the onset of early pachytene stage, ATR phosphorylates histone variant H2AX (γH2AX), which, along with the γH2AX-binding partner MDC1 (Mediator of DNA damage checkpoint protein 1), initiates MSCI [24–26]. Our recent study demonstrated that DDR signaling also sets up escape gene activation through an RNF8 (Ring finger protein 8)-dependent pathway, although RNF8 is not required for gene silencing in MSCI [27]. RNF8 is an E3 ubiquitin ligase that interacts with MDC1 to mediate the somatic DDR [28–30]. During meiosis, RNF8 promotes ubiquitination on the sex chromosomes [31, 32] and, subsequently, establishes a downstream cascade of active epigenetic modifications, which lead to gene activation in spermatids [27]. Notably, histone crotonylation, a recently identified histone modification, is associated with gene activation on the sex chromosomes in spermatids [33], and the chromodomain protein CDYL is a major regulator of histone crotonylation in this context [34].

We have also found that, during meiosis, a germline-specific Polycomb protein, SCML2 (Sex comb on midleg-like 2), suppresses mono-ubiquitination of histone H2A at Lys119 (H2AK119ub) on the sex chromosomes, leading to the activation of a subset of sex-linked genes in spermatids [35]. Since H2AK119ub is mediated by Polycomb repressive complex 1 (PRC1) in the context of gene silencing [36], the removal of H2AK119ub could potentially be an important step for gene activation. SCML2 is also required for formation of open chromatin on the sex chromosomes during meiosis [37]. These results raise the possibility that RNF8 and SCML2 cooperate to establish epigenetic memories during meiosis through two distinct ubiquitin-mediated regulatory pathways, and that the epigenetic memories induce gene activation later in spermatids. However, given the different functions of these pathways in the regulation of ubiquitination, it remains unknown how they function together to regulate ubiquitination and epigenetic modifications on the sex chromosomes.

In this study, we examined the genetic relationship between *Rnf8* and *Scml2* by generating mice with a double knockout of both genes, and we defined the functions of RNF8 and SCML2 in the regulation of ubiquitination. We show that RNF8 and SCML2 cooperate to regulate distinct forms of ubiquitination on the sex chromosomes. Subsequent to ubiquitin regulation, H3K27 acetylation, a marker of active enhancers [38], is established as an essential preparatory step for gene activation in the round spermatid phase. Our results offer fundamental information on the epigenetic programming of the sex chromosomes and provide a paradigm for understanding ubiquitin regulation in the context of gene activation.

## Results

### RNF8 and SCML2 distinctly regulate ubiquitination on the sex chromosomes

RNF8 promotes polyubiquitination of unknown substrates on the sex chromosomes at the onset of the early pachytene stage of meiotic prophase, when homologous chromosomes complete synapsis [27, 35]; subsequently, SCML2 functions in the early-to-mid pachytene transition to remove monoubiquitination of histone H2A (H2AK119ub) from the sex chromosomes [35, 39] (Fig 1A and 1B). To determine how these separate forms of ubiquitin regulation lead to gene activation, we investigated the localization of different forms of ubiquitination on the sex chromosomes during male meiosis via immunofluorescence microscopy. We used an anti-SYCP3 antibody to judge the stages of meiotic prophase, which can be distinguished based on the status of chromosome synapsis ([40]; see Materials and Methods). We began by investigating the signals detected by FK2, a monoclonal antibody that detects both mono- and polyubiquitinated conjugates [41] (Fig 1). By immunostaining with FK2, we anticipated the detection of all ubiquitin signals established on the sex chromosomes. Our detailed analysis revealed that, in wild-type spermatocytes, FK2 signals accumulated on the sex chromosomes beginning in the early pachytene stage, and accumulation persisted through to the early diplotene stage, when homologous autosomes begin to desynapse (Fig 1C). However, FK2 signals were not detected on the sex chromosomes in the late diplotene stages (Fig 1C), suggesting that ubiquitin conjugates are largely removed from the sex chromosomes by the late diplotene stage. Consistent with our previous studies, FK2 signals depended on the presence of RNF8 and were thus absent in spermatocytes from *Rnf8* knockout (*Rnf8*-KO) mice (Fig 1D and [27]). Further, FK2 signals did not change in *Scml2*-KO spermatocytes as compared to wild-type spermatocytes (Fig 1E and [35]). Although SCML2 catalyzes the removal of monoubiquitination, resulting in a likely increase in ubiquitination of the sex chromosomes in *Scml2*-KO spermatocytes, FK2 signals appeared comparable between wild-type and *Scml2*-KO mice. We expect that comparable levels of immunofluorescence signals result from an overall abundance of polyubiquitination.

Because the FK2 antibody allows us to detect many forms of ubiquitination, we sought to determine a possible genetic relationship between *Rnf8* and *Scml2* by testing global ubiquitination in a mouse model deficient for both *Rnf8* and *Scml2*, termed the *Rnf8;Scml2* double knockout (*Rnf8;Scml2-*dKO). *Rnf8;Scml2-*dKO mice had smaller testes than the wild-type, or the *Rnf8*-KO, and were infertile (S1A and S1B Fig). Although *Rnf8;Scml2-*dKO spermatocytes underwent normal chromosomes synapsis (S1C Fig) and did not show meiotic arrest, the *Rnf8;Scml2-*dKO mice appeared to have more profound testicular defects than that of *Rnf8* or *Scml2* single mutants (S1D–S1F Fig). For example, the population of histone H1T (testis-specific histone H1, which begins to accumulate after the mid pachytene stage)-positive differentiated cells were reduced in the *Rnf8;Scml2-*dKO testes (S1F Fig). These results suggest that the functions of RNF8 and SCML2 are largely independent. The severe phenotype of *Rnf8;Scml2-*dKO is unlikely due to a block in spermatogenesis because 96.9% of tubules (n = 65) evinced H1T-positive spermatids (S1G Fig).

If *Rnf8* is solely required to establish all ubiquitin modifications on the sex chromosomes, there should not be any ubiquitination on the sex chromosomes in the *Rnf8;Scml2-*dKO. However, to our surprise, FK2 signals were present on the sex chromosomes of *Rnf8;Scml2-*dKO spermatocytes (Fig 1F), although signal intensities were markedly reduced in comparison to accumulation patterns observed in wild-type spermatocytes. Thus, the phenotype of the *Rnf8;Scml2-*dKO suggests that, while RNF8 mediates polyubiquitination conjugates of unknown substrates, an unknown E3 ligase mediates H2AK119ub independent of RNF8. Given that SCML2 removes H2AK119ub from the sex chromosomes [35, 39], H2AK119ub mediated by an unknown E3 ligase is likely to remain on the sex chromosomes in the absence of SCML2 in *Rnf8;Scml2-*dKO. A schematic of ubiquitin detected by FK2 in each mutant is shown in Fig 1G.

### RNF8-dependent H2AK119ub is removed by SCML2

**Fig 2:**
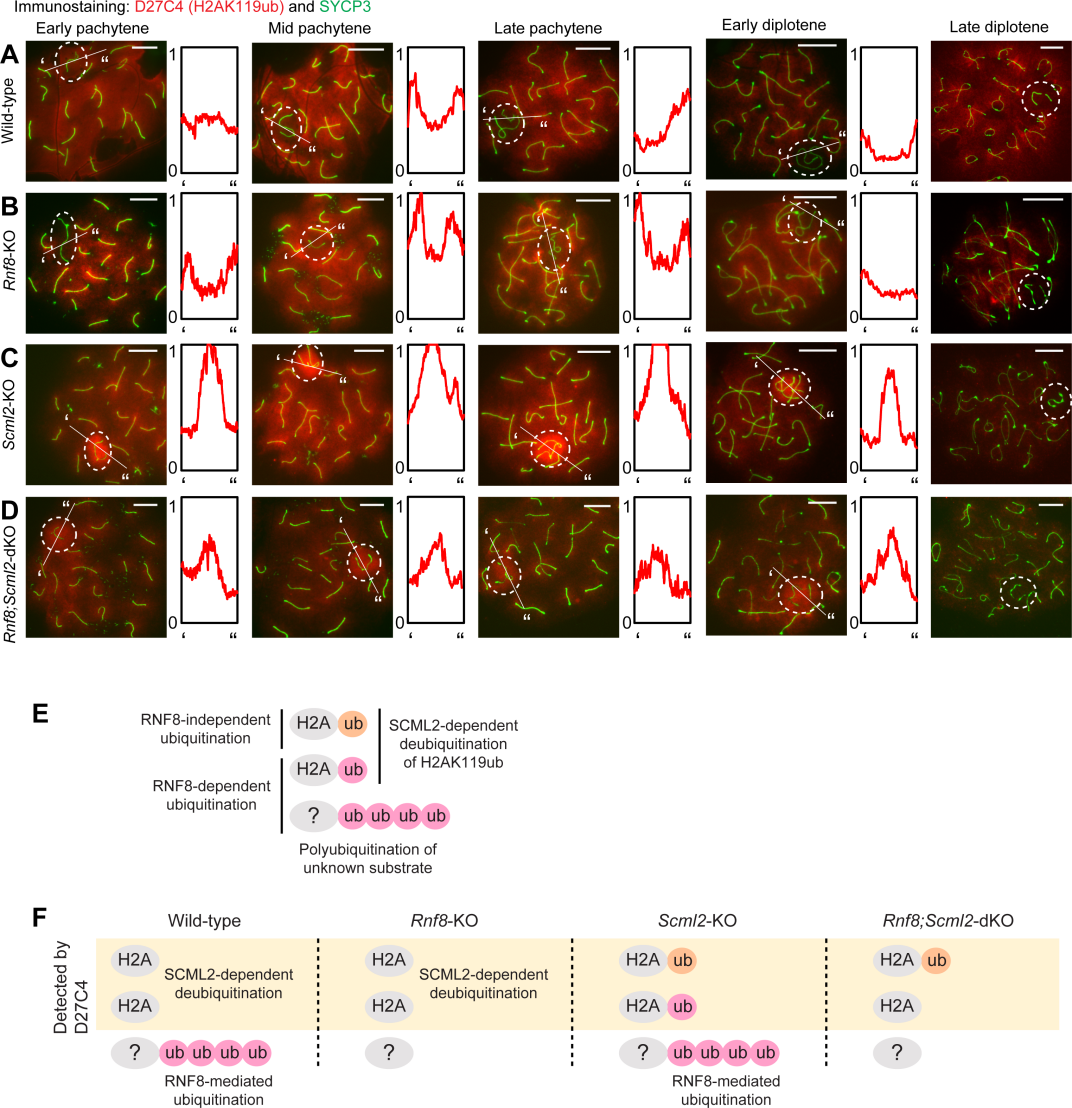
RNF8-dependent H2AK119ub is removed by SCML2: Immunostaining with D27C4 antibody, which recognizes H2AK119ub. (**A-D**) Immunostaining of SYCP3 and D27C4 (H2AK119ub) on meiotic chromosome spreads. Dotted circles: sex chromosomes. Scale bar: 10 μm. Representative images are shown for at least 30 spermatocytes from each substage, from at least 3 independent mice per mouse model. The intensity of immunostaining is quantified by densitometry across the indicated path (‘ to”) and plotted in a relative intensity range of 0–1, which is normalized among all images in this figure, Fig 3 and S2 Fig. (**E**) Updated model of distinct forms of regulation of ubiquitination by RNF8 and SCML2, including RNF8-dependent H2AK119ub, which is removed by SCML2 based on data in this figure. (**F**) Schematic of ubiquitin targets recognized by the D27C4 (H2AK119ub) antibody in each mouse model.

To independently dissect the ubiquitination profiles of the sex chromosomes, we performed immunofluorescence microscopy using a specific monoclonal antibody against H2AK119ub (clone D27C4), which we previously confirmed to recognize H2AK119ub [35]. In wild-type spermatocytes, H2AK119ub signals were present on the sex chromosomes beginning in the early pachytene stage, although H2AK119ub signals on the sex chromosomes decreased through the mid pachytene and subsequent diplotene stages (Fig 2A). Likewise, in *Rnf8*-KO spermatocytes, the H2AK119ub signals decreased on the sex chromosomes; however, the depletion of H2AK119ub signals was evident even in the early pachytene stage, and this decrease continued through subsequent pachytene and diplotene stages (Fig 2B; independent pictures are shown in S2 Fig). This difference suggests that RNF8 is required for the temporal presence of H2AK119ub on the sex chromosomes in early pachytene spermatocytes.

However, in both wild-type and *Rnf8*-KO spermatocytes, the decrease of H2AK119ub occurred on the sex chromosomes in the presence of SCML2; in *Scml2*-KO spermatocytes, an intense H2AK119ub signal accumulated on sex chromosomes starting in the early pachytene stage and persisted until the early diplotene stage (Fig 2C). We explored the genetic relationship between *Rnf8* and *Scml2* by testing the accumulation of H2AK119ub in *Rnf8;Scml2-*dKO spermatocytes. To our surprise, the accumulation of H2AK119ub on the sex chromosomes of *Rnf8;Scml2-*dKO spermatocytes was decreased in comparison to *Scml2*-KO spermatocytes. The *Rnf8;Scml2-*dKO demonstrated an intermediate intensity of H2AK119ub on the sex chromosomes between that of wild-type and *Scml2-*KO beginning in the early pachytene stage and continuing through the early diplotene stage. This intermediate phenotype reveals two distinct forms of H2AK119ub regulation: one is RNF8-dependent H2AK119ub (Fig 2E), and the other is RNF8-independent H2AK119ub mediated by an unknown E3 ligase. Both types of H2AK119ub were detected in the *Scml2*-KO, but only RNF8-independent H2AK119ub was detected in the *Rnf8;Scml2-*dKO (Fig 2F), as demonstrated by the intermediate intensity of H2AK119ub signals observed in the *Rnf8;Scml2-*dKO. Taken together, these results indicate that RNF8 mediates both mono- and polyubiquitination of the sex chromosomes, including H2AK119ub, and SCML2 removes two mechanistically distinct H2AK119ub signals (i.e., RNF8-dependent and RNF8-independent signals) from the sex chromosomes (Fig 2E).

**Fig 3:**
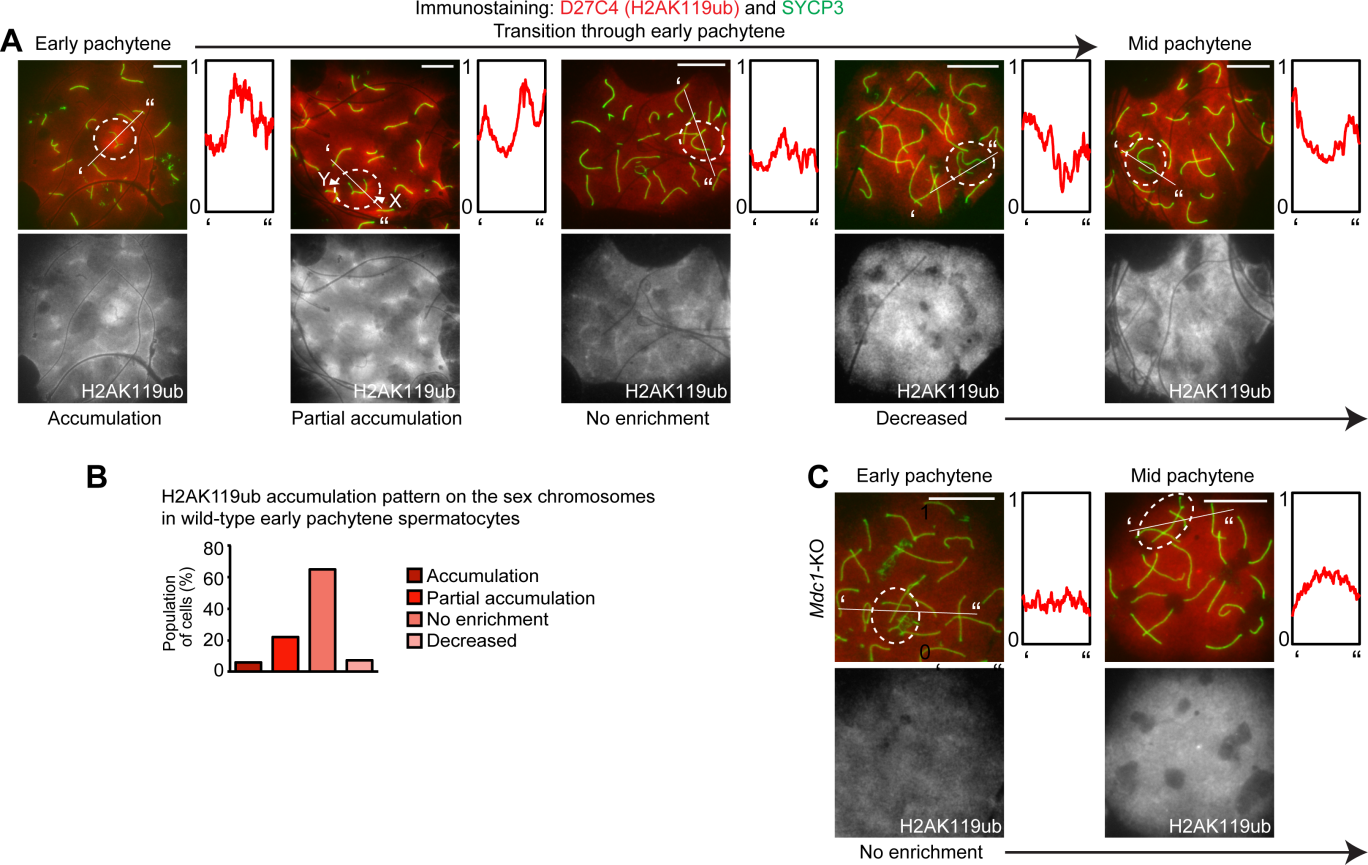
MDC1-dependent H2AK119ub on the sex chromosomes at the onset of the early pachytene stage. (**A)** Immunostaining of SYCP3 and D27C4 (H2AK119ub) on wild-type meiotic chromosome spreads. Dotted circles: sex chromosomes. Scale bar: 10 μm. Representative images are shown for each category through the early pachytene stage for spermatocytes from 9 independent mice. The intensity of immunostaining is quantified by densitometry across the indicated path (‘ to”) and plotted in a relative intensity range of 0–1, which is normalized among all images in this figure, Fig 2 and S2 Fig. (**B**) H2AK119ub accumulation patterns on the sex chromosomes in wild-type early pachytene spermatocytes. A total of 305 early pachytene spermatocytes were scored from 9 independent wild-type mice. (**C**) Immunostaining of SYCP3 and D27C4 (H2AK119ub) on *Mdc1*-KO meiotic chromosome spreads. H2AK119ub accumulation was not observed in *Mdc1*-KO spermatocytes. Dotted circles: sex chromosomes. Scale bar: 10 μm. Representative images are shown through the early pachytene stage for spermatocytes from 3 independent *Mdc1*-KO mice. The intensity of immunostaining is quantified by densitometry across the indicated path (‘ to”) and plotted in a relative intensity range of 0–1, which is normalized among all images in this figure, Fig 2 and S2 Fig.

Since we found that the timing of the H2AK119ub signal decrease was different between wild-type and *Rnf8*-KO spermatocytes in the early-to-mid pachytene transition, we carefully dissected the decrease of H2AK119ub in the early pachytene stage of wild-type spermatocytes. Surprisingly, we observed dynamic changes in H2AK119ub on the sex chromosomes during this relatively brief window of meiotic prophase. In a rare population, H2AK119ub accumulated on the entire domain of sex chromosomes (“Accumulation” in Fig 3A), followed by a decrease of the signal in a stepwise fashion. H2AK119ub started to disappear from the Y chromosome but remained on the X chromosome (“Partial accumulation” in Fig 3A). Next, the level of H2AK119ub became comparable between the sex chromosomes and autosomes (“No enrichment” in Fig 3A), and a decrease in H2AK119ub relative to autosomes occurred by the end of the early pachytene stage (“Decreased” in Fig 3A). Population analysis revealed that the accumulation of H2AK119ub was transient at the beginning of the early pachytene stage (Fig 3B). Because we found the accumulation of H2AK119ub at the very beginning of the early pachytene stage, we tested whether this H2AK119ub is established downstream of MDC1, an interacting partner of RNF8 that directs chromosome-wide spreading of γH2AX to initiate MSCI at the onset of the early pachytene stage [25]. We previously showed that RNF8 functions downstream of MDC1 in male meiosis because RNF8-mediated FK2 signals on the sex chromosomes are abolished in *Mdc1*-KO spermatocytes [25]. Further analysis of *Mdc1*-KO spermatocytes found that MDC1 is required for the accumulation of H2AK119ub (Fig 3C). Because MDC1 is required for the chromosome-wide domain formation of DNA damage signaling on the sex chromosomes, thereby initiating meiotic sex chromosome inactivation [25], there is no chromosome-wide domain formation of the sex chromosomes in *Mdc1*-KO spermatocytes (Fig 3C). Together with the data showing RNF8-dependent H2AK119ub in the early pachytene stage (Fig 2B), these results demonstrate that the chromosome-wide establishment of H2AK119ub occurs downstream of MDC1 and RNF8 on sex chromosomes at the onset of the early pachytene stage.

**Fig 4:**
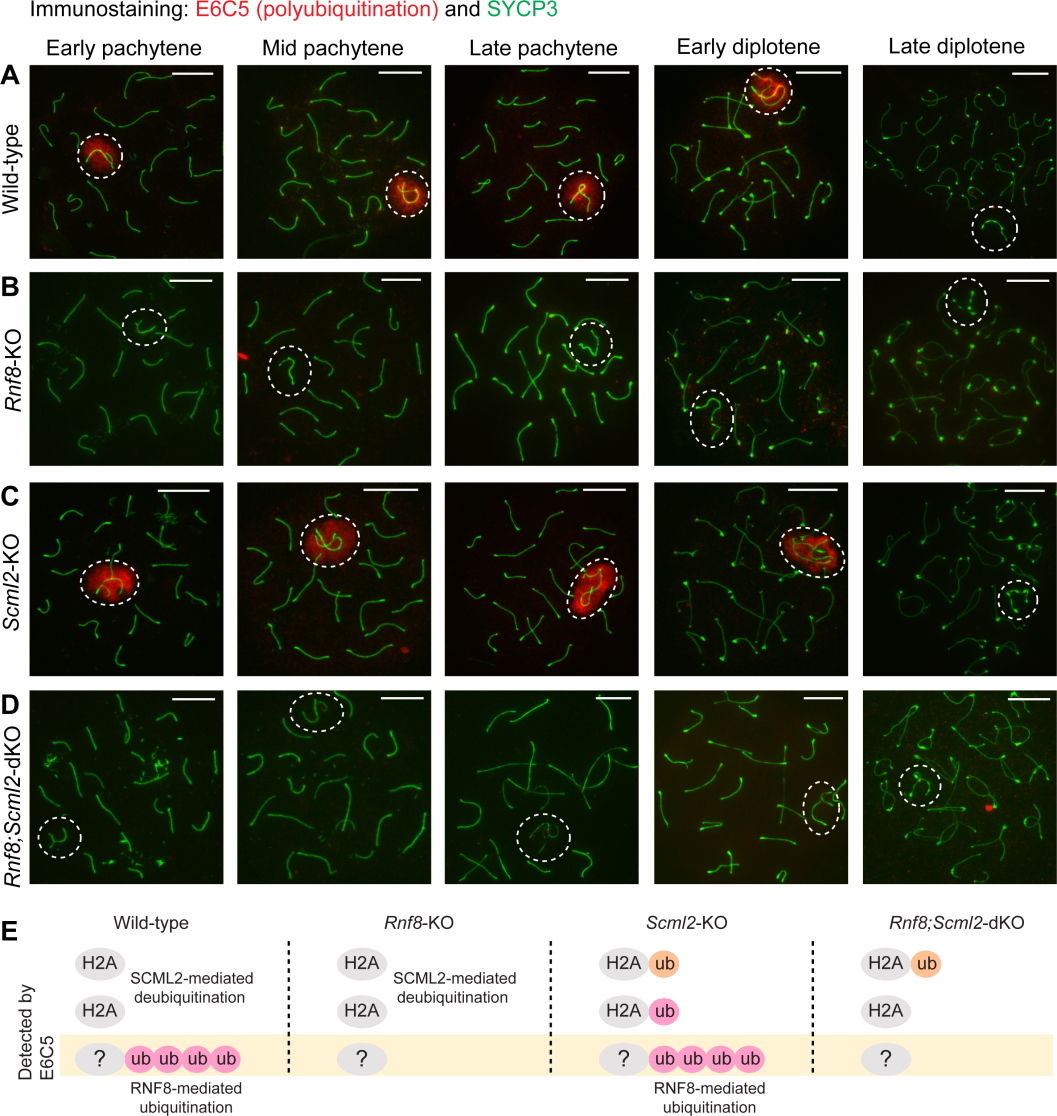
RNF8 is required for polyubiquitination of the sex chromosomes: Immunostaining with E6C5 antibody, which recognizes polyubiquitination. (**A-D**) Immunostaining of SYCP3 and E6C5 on meiotic chromosome spreads. Dotted circles: sex chromosomes. Scale bar: 10 μm. Representative images are shown for at least 30 spermatocytes from each substage, from at least 3 independent mice per mouse model. (**E**) Schematic of ubiquitin targets recognized by the E6C5 antibody in each mouse model.

To confirm our conclusion, we performed additional immunostaining using another monoclonal antibody (clone E6C5) confirmed to recognize polyubiquitination of unknown substrates and not H2AK119ub [35]. As polyubiquitination is mediated by RNF8 (Fig 4A and 4B) and is independent of SCML2 (Fig 4C) [35], we observed no accumulation of polyubiquitination on the sex chromosomes in the *Rnf8;Scml2-*dKO spermatocytes (Fig 4D). Therefore, based on the results from our four mouse models, our findings support our conclusion that polyubiquitination is exclusively mediated by RNF8 and not by SCML2 (Fig 4E).

Taken together, our results clarify the functional relationship between RNF8 and SCML2 in the regulation of ubiquitination on the sex chromosomes, and we demonstrate that RNF8 has a previously unrecognized role in mediating H2AK119ub on the sex chromosomes. Further, while the functions of RNF8 and SCML2 are largely independent, they are, in part, functionally connected through the removal of RNF8-dependent H2AK119ub by SCML2 (Fig 2E).

### RNF8 mediates efficient recruitment of SCML2 to the sex chromosomes

**Fig 5:**
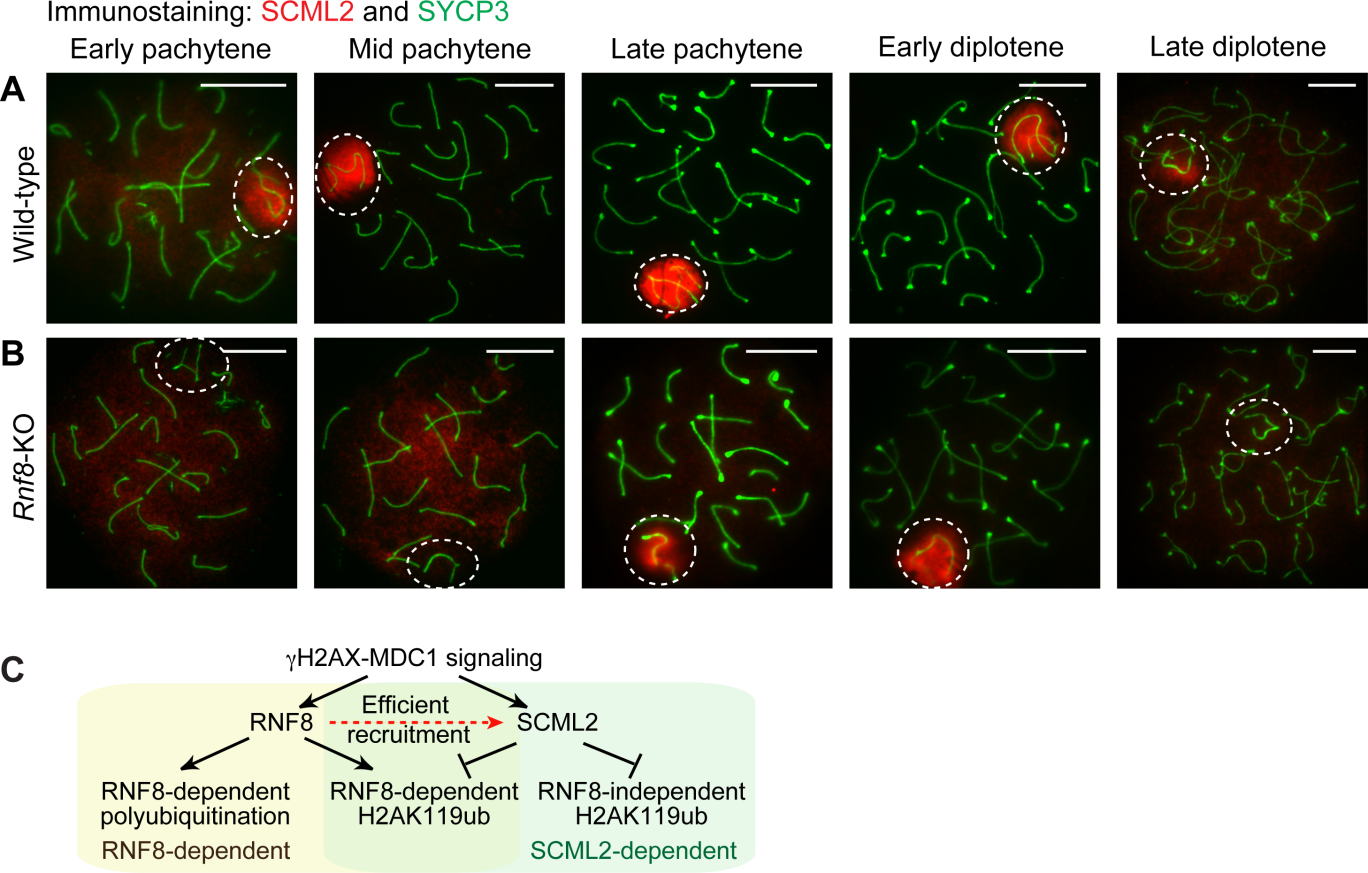
RNF8 is involved in the efficient recruitment of SCML2 to the sex chromosomes. (**A, B**) Immunostaining of SYCP3 and SCML2 on meiotic chromosome spreads. Dotted circles: sex chromosomes. Scale bar: 10 μm. Representative images are shown for at least 30 spermatocytes from each substage, from at least 3 independent mice per mouse model. (**C**) Model of the functions of RNF8 and SCML2 in the regulation of the ubiquitin network.

On the sex chromosomes, both RNF8 and SCML2 function downstream of a DDR pathway centered on γH2AX and MDC1 [27, 35]. Given that RNF8 is an interacting partner of MDC1, and given that SCML2 is recruited to the sex chromosomes during the early-to-mid pachytene transition after the establishment of RNF8-dependent ubiquitination [35, 39], we sought to clarify the involvement of RNF8 in the recruitment of SCML2 to the sex chromosomes (Fig 5A). In *Rnf8*-KO spermatocytes, the recruitment of SCML2 to the sex chromosomes was delayed: accumulation occurred not in the early pachytene stage, as in wild-type spermatocytes (Fig 5A), but during the late pachytene stage (Fig 5B). Furthermore, SCML2 accumulation on the sex chromosomes disappeared in the late diplotene stage of *Rnf8*-KO spermatocytes, in contrast to persistent accumulation on wild-type sex chromosomes. These results suggest that RNF8 is involved in the efficient recruitment and stability of SCML2 on the sex chromosomes during meiosis. In concert with the ubiquitination analysis above, our results establish a hierarchy of pathways, centered on RNF8 and SCML2, that act downstream of γH2AX-MDC1 signaling for the regulation of ubiquitination on the sex chromosomes (Fig 5C).

### RNF8 and SCML2 cooperate to regulate H3K4me2 on the sex chromosomes

**Fig 6:**
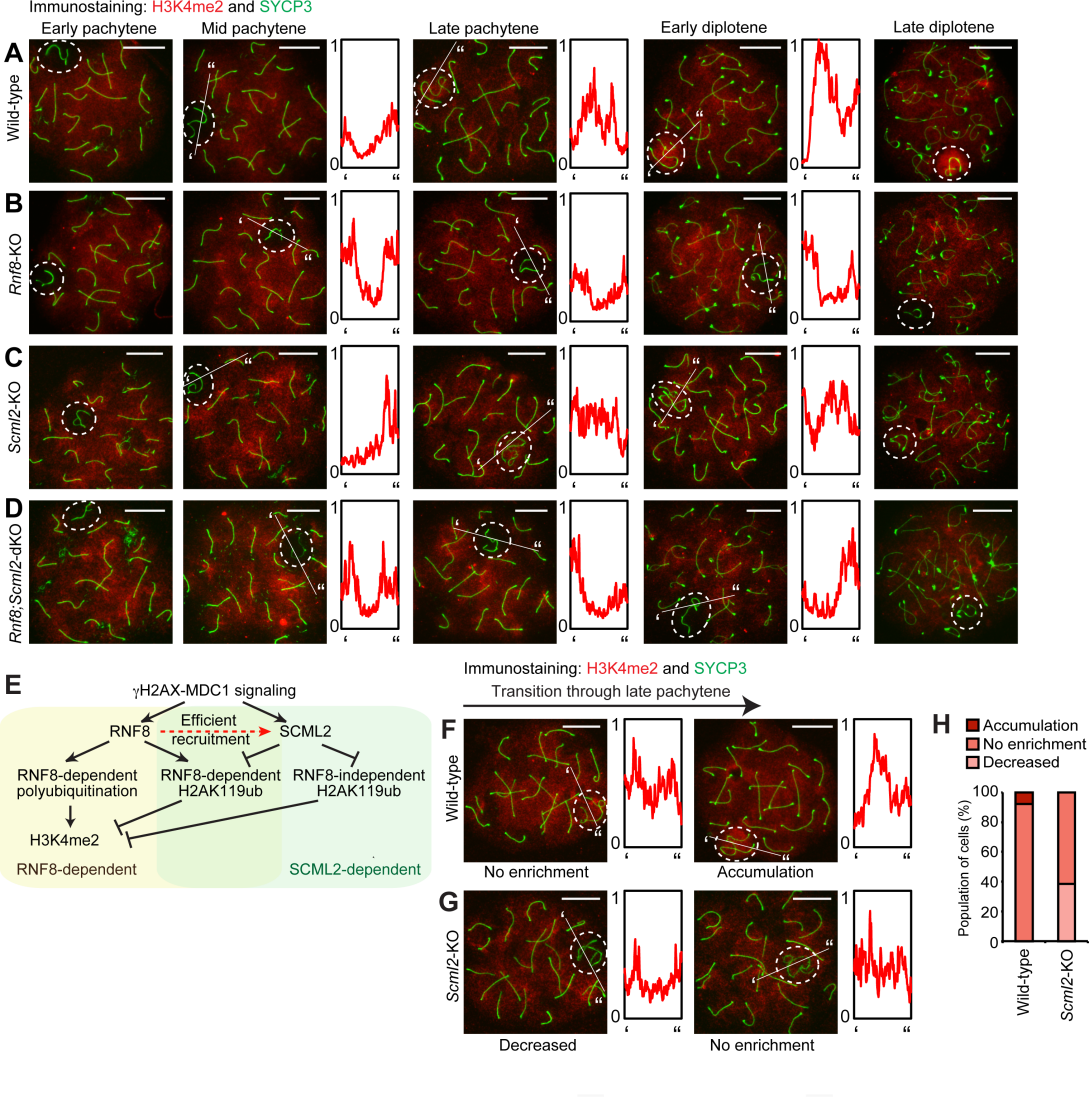
RNF8-dependent establishment of H3K4me2 and its inhibition by H2AK119ub. (**A-D**) Immunostaining of SYCP3 and H3K4me2 on meiotic chromosome spreads. Dotted circles: sex chromosomes. Scale bar: 10 μm. Representative images are shown for at least 30 spermatocytes from each substage, from at least 3 independent mice per mouse model. The intensity of immunostaining is quantified by densitometry across the indicated path (‘ to”) and plotted in a relative intensity range of 0–1, which is normalized among all pictures in this figure. (**E**) Model of the regulation of H3K4me2. (**F, G**) Immunostaining of SYCP3 and H3K4me2 on wild-type and *Scml2*-KO meiotic chromosome spreads. Dotted circles: sex chromosomes. Scale bar: 10 μm. Representative images are shown for late pachytene spermatocytes from 6 independent wild-type mice and 3 independent *Scml2*-KO mice. The intensity of immunostaining is quantified by densitometry across the indicated path (‘ to”) and plotted in a relative intensity range of 0–1, which is normalized among all pictures in this figure. (**H**) H3K4me2 accumulation patterns on the sex chromosomes in late pachytene spermatocytes. A total of 244 late pachytene spermatocytes were scored from 6 independent wild-type mice, and a total 91 late pachytene spermatocytes were scored from 3 independent *Scml2*-KO mice.

We next sought to dissect how this ubiquitin regulatory network coordinates downstream active epigenetic modifications for escape gene activation. Since RNF8 is required to establish dimethylation of H3K4 (H3K4me2) [27], an active epigenetic modification, we examined the accumulation dynamics of H3K4me2 downstream of the ubiquitin regulatory network. In wild-type spermatocytes, H3K4me2 was depleted from the sex chromosomes at the onset of the pachytene stage; however, we observed a gradual establishment of H3K4me2 in the late pachytene stage, and this accumulation persisted into the later stages of diplotene (Fig 6A). In both *Rnf8*-KO and *Rnf8;Scml2-*dKO spermatocytes, H3K4me2 was depleted from the sex chromosomes throughout meiotic prophase (Fig 6B and 6D), indicating that RNF8 is necessary for the establishment of H3K4me2. Interestingly, in *Scml2*-KO spermatocytes, the establishment of H3K4me2 in the late pachytene stage never surpassed an intermediate level of signal intensity, less than that in wild-type spermatocytes but greater than the depletion observed in *Rnf8*-KO and *Rnf8;Scml2*-dKO spermatocytes (Fig 6C). This intermediate level of accumulation intensity was maintained through the diplotene stages of *Scml2-*KO spermatocytes (Fig 6C). Together with our ubiquitination analysis, these results suggest that RNF8-mediated polyubiquitination is required for the establishment of H3K4me2, while H2AK119ub inhibits the establishment of H3K4me2 (Fig 6E).

Intrigued by the intermediate signal intensity of H3K4me2 in SCML2-deficient spermatocytes, we carefully detailed the accumulation dynamics of H3K4me2 in late pachytene spermatocytes from our wild-type and *Scml2*-KO models. Our analysis revealed a delay in the establishment of H3K4me2 in *Scml2-*KO spermatocytes (Fig 6F and 6G). Because of the gradual accumulation of H3K4me2 on the sex chromosomes, H3K4me2 intensity on the sex chromosomes reached a similar level to that on the autosomes in 90% of wild-type late pachytene spermatocytes (Fig 6F and 6H). The remaining 10% of nuclei showed clear enrichment of H3K4me2 signals on the sex chromosomes (Fig 6F and 6H). However, in the *Scml2-*KO spermatocytes, H3K4me2 signals remained at a lower level on the sex chromosomes relative to autosomes in 40% of late pachytene spermatocytes (“Decreased” in Fig 6G and 6H); in the other 60% of late spermatocytes, the level of H3K4me2 intensity was increased on sex chromosomes, reaching an intensity similar to that of autosomes (“No enrichment” in Fig 6G and 6H). Therefore, these results suggest that the SCML2-dependent removal of H2AK119ub facilitates RNF8-dependent establishment of H3K4me2.

### RNF8 and SCML2 cooperate to regulate H3K27ac prior to H3K4me2 accumulation on the sex chromosomes

While H3K4me2 is established on the sex chromosomes during meiosis, actual gene activation of escape genes occurs in the postmeiotic round spermatid stage [27], suggesting that escape genes are poised for activation during meiosis. Since H3K4me2 accumulates on the promoters of poised genes during spermatogenesis [42], we sought to determine whether enhancers on the sex chromosomes are similarly poised during meiosis for escape gene activation in round spermatids. To test this, we investigated the localization of acetylation of H3K27 (H3K27ac), a marker of active enhancers [38], during meiosis.

**Fig 7:**
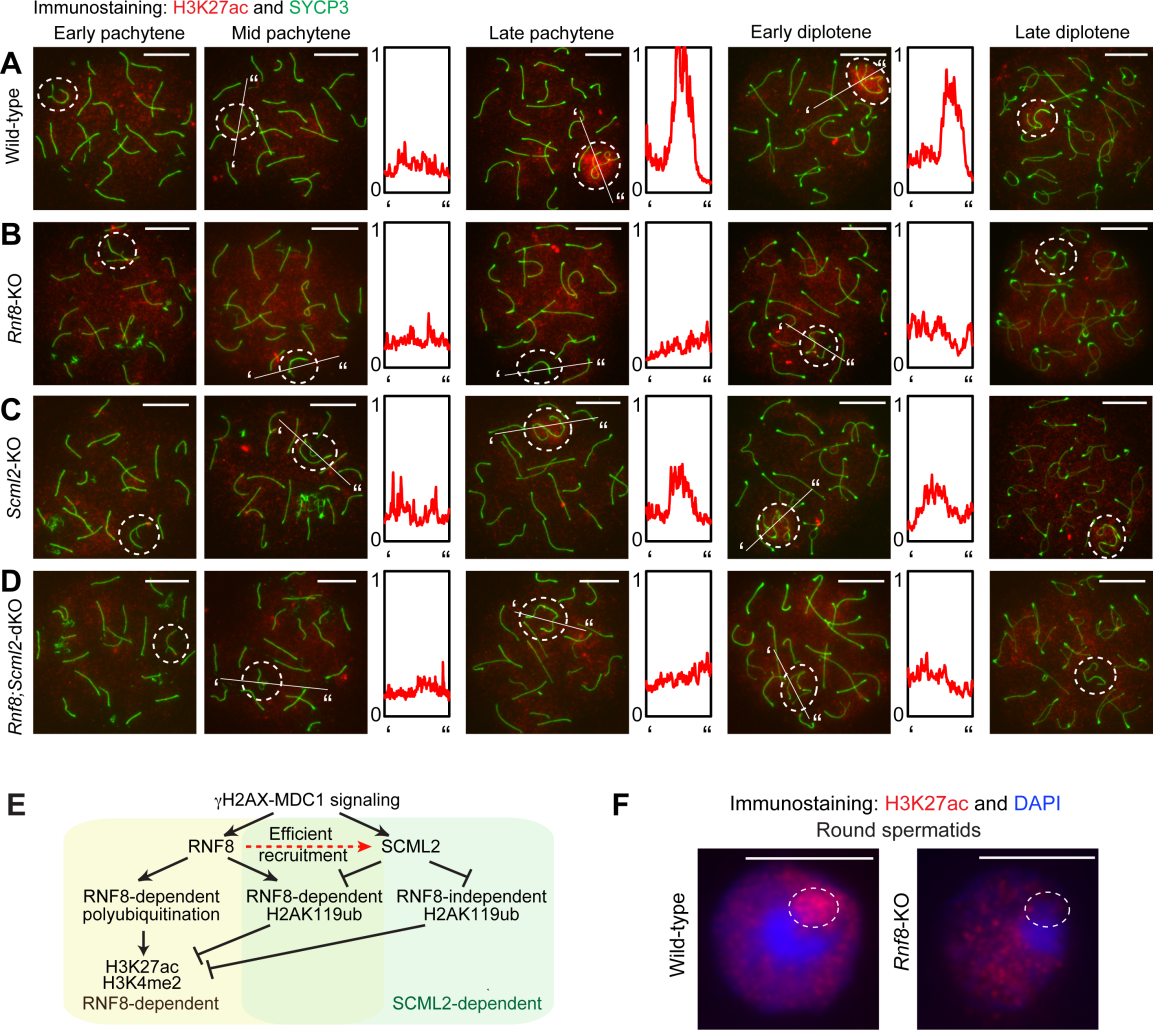
RNF8-dependent establishment of H3K27ac and its inhibition by H2AK119ub. (**A-D**) Immunostaining of SYCP3 and H3K27ac on meiotic chromosome spreads. Dotted circles: sex chromosomes. Scale bar: 10 μm. Representative images are shown for at least 30 spermatocytes from each substage, from at least 3 independent mice per mouse model. The intensity of immunostaining is quantified by densitometry across the indicated path (‘ to”) and plotted in a relative intensity range of 0–1, which is normalized among all pictures in **A-D**. (**E**) Model of the regulation of H3K27ac. (**F**) Immunostaining of SYCP3 and H3K27ac on wild-type and *Rnf8*-KO round spermatids using slides that preserve the relative nuclear organization of spermatogenic cells. Dotted circles: post-meiotic sex chromatin. Scale bar: 10 μm. Representative images are shown for at least 30 round spermatids from at least 3 independent mice.

In wild-type spermatocytes, H3K27ac accumulated on the sex chromosomes in the late pachytene stage (Fig 7A) and persisted there through the early diplotene stage, after which the intensity rapidly decreased through the late diplotene stage. However, in *Rnf8*-KO spermatocytes, H3K27ac accumulation on the sex chromosomes was largely depleted throughout meiotic prophase (Fig 7B), indicating that RNF8 is required for the establishment of H2K27ac on the sex chromosomes during meiosis. In *Scml2*-KO spermatocytes, H2K27ac accumulated on the sex chromosomes in the late pachytene stage, as seen by comparison to signals on autosomes, but the intensity of H3K27ac was at an intermediate level between the signal intensities observed on wild-type and *Rnf8*-KO sex chromosomes (Fig 7C). Therefore, it is possible that H2AK119ub must be removed for efficient establishment of H3K27ac, as is the case for H3K4me2. Finally, in *Rnf8;Scml2-*dKO spermatocytes, H3K27ac did not accumulate on the sex chromosomes. These results indicate that RNF8 is essential for H3K27ac accumulation. Taken together, our data reveal that the regulation of H3K27ac occurs in the same pathway as the regulation of H3K4me2, where RNF8 plays an essential role and H2AK119ub is inhibitory to the establishment of the active epigenetic modifications (Fig 7E).

Importantly, the accumulation of H3K27ac was fully established in the late pachytene stage of wild-type spermatocytes prior to the establishment of H3K4me2 (Figs 6A and 7A). Thus, we infer that poised enhancers marked with H3K27ac are organized prior to the formation of poised promoters marked with H3K4me2. Notably, H3K27ac persisted on sex chromosomes into the postmeiotic round spermatid stage and was detected on postmeiotic sex chromatin (PMSC; Fig 7F), a silent chromatin compartment housing either of the two male sex chromosomes [20]. In round spermatids of the *Rnf8;Scml2-*dKO, the DAPI-dense structure of PMSC is present but H3K27ac was depleted from the PMSC (S3 Fig). Therefore, H3K27ac may serve as a persistent epigenetic memory established during meiosis, maintained through meiotic divisions, and translated to gene activation days after its initial accumulation.

### RNF8 and SCML2 cooperatively activate escape genes on the sex chromosomes in round spermatids

Since we determined the regulatory pathways for H3K4me2 and H3K27ac, we next sought to describe the genomic distribution of these modifications on the sex chromosomes using the chromatin immunoprecipitation with sequencing (ChIP-seq) assay. We analyzed two representative stages of spermatogenesis: (1) to identify the establishment of H3K4me2 and H3K27ac modifications, we analyzed pachytene spermatocytes (PS) purified from adult testes; and (2) to determine the persistence of these modifications in postmeiotic stages, we analyzed round spermatids (RS) purified from adult testes. For the analysis of H3K4me2, we used our publish data [42], and for the analysis of H3K27ac, we carried out ChIP-seq for two independent biological replicates, confirming high levels of reproducibility between the replicates (S4A Fig).

**Fig 8:**
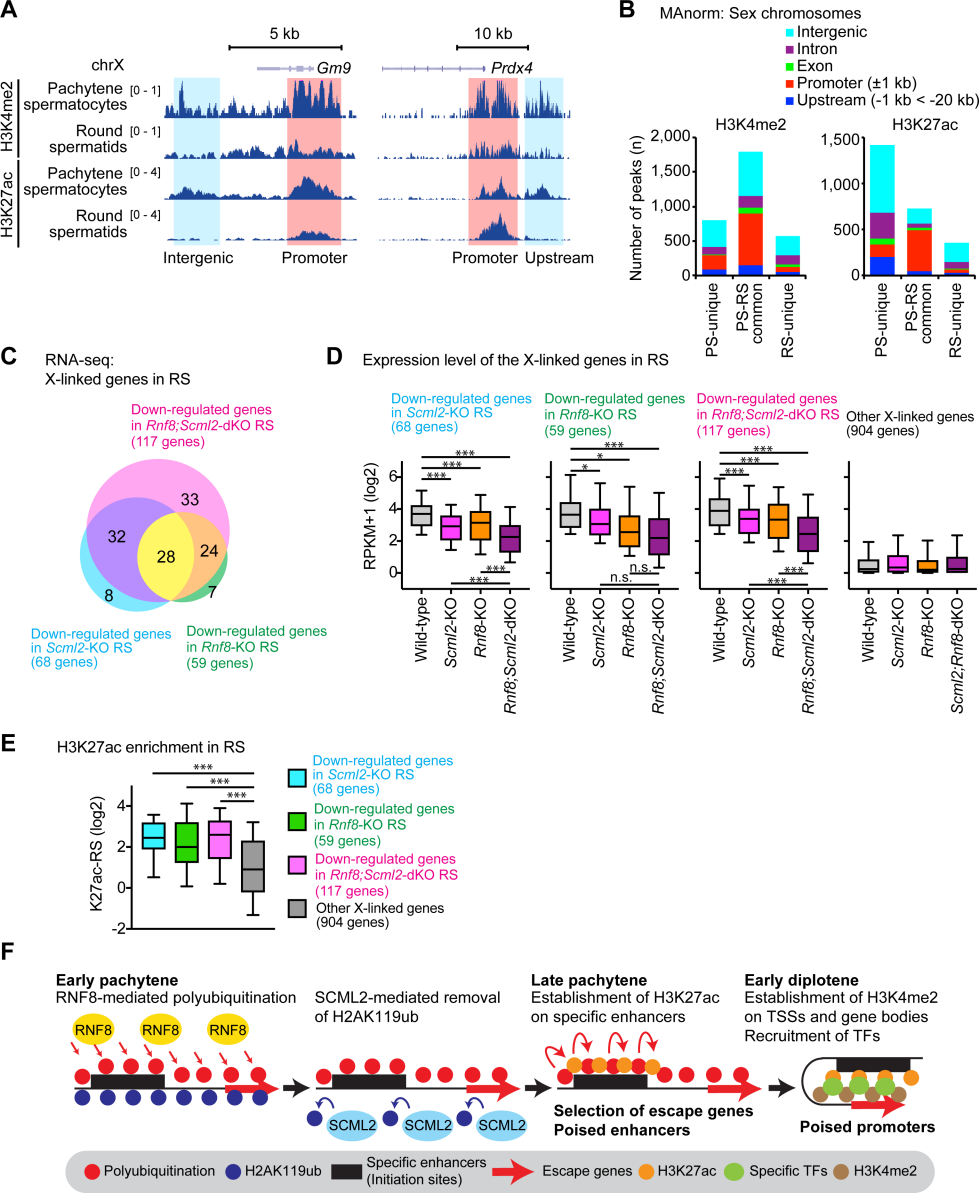
Genomic distribution of H3K4me2 and H3K27ac on the sex chromosomes in late spermatogenesis. (**A**) Track view of ChIP-seq data with biological replicates in pachytene spermatocytes (PS) and round spermatids (RS) of wild-type mice. (**B**) MAnorm analysis of ChIP-seq peaks of H3K4me2 and H3K27ac in PS and RS of wild-type mice. The genomic distribution of each peak is shown with colored bars. (**C**) The number of down-regulated genes in mutant round spermatids detected by RNA-seq (more than 1.5 fold change, expression in wild-type RS is at least 3 RPKM, and *P*_adj_ is less than 0.05) between wild-type and each mutant (two biological replicates). (**D**) Expression levels of X-linked genes for each class in RS of the indicated genotypes. Other genes include all X-linked genes that are not classified into the three groups. * *P* < 0.01, *** *P* < 0.0001, paired t-test. (**E**) Enrichment analysis of H3K27ac (ChIP-seq) around ±2 kb from transcription starts sites for each class of genes. * *P* < 0.01, *** *P* < 0.0001, paired t-test. (**F**) Schematic model of gene selection mechanisms for gene activation. Specific enhancers serve as initiation sites by which escape genes can be selected.

In PS, we found that H3K4me2 and H3K27ac were associated with promoter and intergenic regions of the *Gm9* and *Prdx4* loci, which both represent a class of X-linked RNF8-dependent escape genes (Fig 8A). For the quantitative analyses of ChIP-seq peaks between PS and RS, we used the peak analysis software MAnorm to compare peaks derived from two pairwise next-generation sequencing datasets [43]. On the sex chromosomes, H3K4me2 peaks tend to be distributed in promoter and intergenic regions, and this distribution is maintained from PS into postmeiotic stages (Fig 8B). On the other hand, we detected many H3K27ac peaks unique to intergenic and intronic regions on the sex chromosomes in PS, consistent with its role at enhancers, and only a portion of these peaks persisted into RS (Fig 8B). H3K4me2 and H3K27ac peaks on the sex chromosomes largely overlapped each other at promoter regions, while many H3K27ac peaks unique to intergenic and intronic regions on the sex chromosomes did not overlap with H2K4me2 peaks (S4B Fig). The establishment and persistence of these modifications from PS to RS suggest that H3K4me2 and H3K27ac act on the regulatory regions of sex chromosomes for escape gene activation.

To determine the set of escape genes regulated by both SCML2 and RNF8, we performed RNA-sequencing (RNA-seq) using PS and RS purified from the *Rnf8;Scml2-*dKO. The RNA-seq results of *Rnf8;Scml2-*dKO cells (two-biological replicates) were analyzed with our previous data obtained from wild-type, *Rnf8-*KO, and *Scml2-*KO mice [35, 42]. To identify differentially expressed genes, we applied the following criteria: genes evincing a >1.5-fold change in expression, expression in wild-type RS ≥3 RPKM, and *P*_adj_ < 0.05. Although gene expression profiles remained largely unchanged in wild-type and mutant PS, escape genes were largely down-regulated in the RS of *Rnf8;Scml2-*dKO (S1 and S2 Tables). 68 genes were down-regulated in *Scml2-*KO RS, 59 genes were down-regulated in *Rnf8-*KO RS, and among those, 28 genes were commonly down-regulated in the RS of both the *Rnf8-KO* and *Scml2-*KO (Fig 8C, S3 Table). Many of these genes were down-regulated in the *Rnf8;Scml2-*dKO RS, while 33 genes were exclusively down-regulated in *Rnf8;Scml2-*dKO RS. Next, we investigated the expression levels of groups of X-linked genes to determine the nature of escape gene regulation by both RNF8 and SCML2. The group of genes down-regulated in *Scml2*-KO RS were also down-regulated in *Rnf8*-KO RS, and vice versa: the group of genes down-regulated in *Rnf8*-KO RS were also down-regulated in *Scml2*-KO RS (Fig 8D). Both groups of genes were further down-regulated in the RS of the *Rnf8;Scml2-*dKO (Fig 8D). These results demonstrate that the functions of RNF8 and SCML2—while largely independent—converge to cooperatively activate escape genes on the X chromosome in RS.

We previously demonstrated that H3K4me2 is enriched on escape genes in RS in an RNF8-dependent manner, while H3K4me2 is also enriched on X-linked genes repressed in meiotic and post-meiotic cells [42]. These genes, which are subject to postmeiotic silencing, are proposed to be poised for activation after fertilization [42]. Thus, H3K4me2 is associated with both escape gene activation and gene poising during postmeiotic silencing. To further define the mechanism for escape gene activation, we sought to determine whether H3K27ac accumulates on escape genes more than on non-escape genes on the RS X chromosome. Indeed, H3K27ac is highly enriched on escape genes that are regulated by RNF8 and SCML2 in comparison to other genes that are regulated by neither RNF8 nor SCML2 (Fig 8E). Therefore, these results suggest that H3K27ac, like H3K4me2, is associated with escape gene activation in RS.

## Discussion

In this study, we elucidate the genetic relationship between RNF8 and SCML2, two regulatory factors necessary for escape gene activation [27, 35]. We also illuminate a ubiquitin regulatory network that facilitates the deposition of active histone modifications on the sex chromosomes during meiosis for postmeiotic escape gene activation. Using three independent antibodies that recognize different forms of ubiquitination, we found that SCML2 facilitates the removal of RNF8-dependent H2AK119ub, demonstrating an important form of interplay between RNF8 and SCML2 (Fig 2E). Interestingly, SCML2 also facilitates the removal of RNF8-independent H2AK119ub, and we also confirmed the presence of RNF8-dependent polyubiquitination that is not deubiquitinated in an SCML2-dependent manner. Considering how they are jointly regulated by RNF8 and/or SCML2, polyubiquitination, RNF8-dependent H2AK119ub, and RNF8-independent H2AK119ub are distinct from each other.

H2AK119ub is a characteristic mark mediated by RNF2, which is a major catalytic subunit of PRC1 [36]. In somatic cells, DNA damage triggers RNF2-dependent H2AK119ub (monoubiquitination) [44]. Therefore, it is possible that RNF8-independent H2AK119ub during meiosis is mediated by RNF2 downstream of γH2AX-MDC1 signaling. Furthermore, in somatic cells, RNF8 mediates both mono- and polyubiquitination of H2A and H2AX [29, 30]. Our identification of RNF8-dependent H2AK119ub on the sex chromosomes reveals a commonality between the somatic DDR pathway and the DDR pathway on sex chromosomes. This finding further supports the notion that DNA damage response pathways are adapted to regulate the sex chromosomes during meiosis [25]. Based on work presented here, we conclude that two distinct forms of regulation for H2AK119ub (RNF8-independent and RNF8-dependent) take place downstream of γH2AX-MDC1 signaling on the chromosome-wide domain of the sex chromosomes.

While MDC1 recruits RNF8 in the somatic DDR [28–30], these proteins appear to have largely distinct functions during male meiosis. In contrast to MDC1, which promotes chromosome-wide silencing of the sex chromosomes during meiosis [25], RNF8 instead promotes the expression of escape genes [27]. Still, we find that both MDC1 and RNF8 are required for H2AK119ub on the chromosome-wide domain of the sex chromosomes, and therefore may cooperate in this process, perhaps analogous to the cooperation of MDC1 and RNF8 in mediating polyubiquitination/monoubiquitination of H2A and H2AX during the somatic DDR. A cooperative role of MDC1 and RNF8 in mediating histone ubiquitination may therefore represent its “primordial” function since it appears to be shared between the somatic DDR and male meiosis.

We also find that RNF8 promotes, but is not required for, the accumulation of SCML2 on the sex chromosomes. Since SCML2 accumulation is delayed and attenuated, but not abrogated in the *Rnf8*-KO (Fig 5), the accumulation of SCML2 may not be due to a physical interaction between these proteins. Perhaps RNF8-mediated ubiquitination of chromatin, whether in the form of polyubiquitination or monoubiquitination, or both, creates an environment that promotes the accumulation of SCML2. Although SCML2 accumulates on the sex chromosomes during the transition between early-to-mid pachytene stages, we found that the abnormal H2AK119ub signals were obvious in the early pachytene stage of *Scml2*-KO mice. Therefore, these results suggest that SCML2’s function does not necessarily reflect its accumulation status on the sex chromosomes.

With the analysis of active histone modifications, we reveal the functional significance of a ubiquitin network on the sex chromosomes. RNF8 is required to establish H3K27ac and H3K4me2, and deubiquitination of H2K119ub by SCML2 appears to be necessary for levels commensurate with the accumulation of H3K27ac and H3K4me2 on wild-type sex chromosomes. There are at least two possible mechanisms for the regulation of H3K27ac and H3K4me2 active marks. First, as we propose in a model described in Figs 6E and 7E, RNF8-dependent polyubiquitination may establish H3K27ac and H3K4me2, while the presence of H2AK119ub is inhibitory to these modifications. Since RNF8 mediates polyubiquitination of unknown substrates during meiosis, identifying theses substrates is an important next step to dissect the link between DDR pathways and the establishment of active epigenetic modifications. The second possibility is that RNF8-dependent H2AK119ub is involved in establishing H3K27ac and H3K4me2, perhaps in conjunction with RNF8-dependent polyubiquitination events. Subsequently, SCML2-dependent deubiquitination of H2AK119ub might promote the normal accumulation of H3K27ac and H3K4me2 marks. This second model is based upon the possibility that completion of a ubiquitination-deubiquitination cycle at H2AK119 regulates active marks on H3. Since SCML2 is recruited after initial RNF8-dependent ubiquitination of chromatin on the sex chromosomes, subsequent deubiquitination of H2AK119 by SCML2 could provide a mechanism that controls the timing of H3K27ac and H3K4me2 accumulation.

Based on our data, we propose that there are two critical steps for escape gene activation: the first step is epigenetic programming that establishes active epigenetic memories on silent sex chromosomes during meiosis, and the second step occurs after meiotic division when genes are activated in spermatids based on established epigenetic memories. Here, we identify H3K4me2, a modification associated with active transcription [45], and H3K27ac, a marker of active enhancers [38], as candidate factors for epigenetic memories that persist from meiosis to spermatids.

The identification of regulatory mechanisms and the genomic distribution of two active epigenetic modifications, H3K4me2 and H3K27ac, allow us to speculate how escape genes are targeted for activation via RNF8- and SCML2-dependent mechanisms. Since H3K27ac establishment precedes H3K4me2 establishment on the sex chromosomes, it is possible that specific enhancers serve as initiation sites by which escape genes can be selected (Fig 8C). Our cytological analysis reveals that RNF8- and SCML2-dependent modifications occur in a coordinated manner during specific substages of meiotic prophase. RNF8 mediates ubiquitination while SCML2 removes H2AK119ub in the early pachytene stage, and H3K27ac appears on the sex chromosome in the late pachytene stage. We therefore speculate that intrinsic genomic and epigenomic features of escape gene enhancers provide the initial mark(s) that allow(s) escape genes to be selected for RNF8- and/or SCML2-dependent epigenetic modification in the early pachytene stage. We speculate that specific transcription factors (TFs) and H3K4me2 are then established on escape genes during the pachytene-to-diplotene transition. This is a new view of how escape genes are activated downstream of a chromosome-wide ubiquitin regulatory network on the sex chromosomes, and the identification and functional determination of enhancers marked by H3K27ac makes for an intriguing follow-up goal.

Although this study is based on mouse models, the findings are relevant to key issues pertaining to human male infertility, specifically the regulation of sex-linked genes essential for sperm development. Our study provides fundamental information regarding epigenetic programming and serves as a general paradigm for epigenetic gene activation. The essential epigenetic mechanism of sex-linked gene expression is highly conserved in human and mouse spermatogenesis [19]. Thus, elucidating a gene activation mechanism in a mouse model should directly increase our knowledge of human male infertility.

## Materials and methods

### Animals and breeding

*Rnf8-* and *Scml2*-knockout (KO) mice are described in the literature [35, 46]. Both mouse models are on a C57BL/6 background. Heterozygous *Rnf8* males and females were bred to produce *Rnf8*-KO male pups. Because *Scml2* is an X-linked gene, heterozygous *Scml2* females were bred with wild-type C57BL/6 male mice to produce *Scml2*-KO male pups. Heterozygous *Rnf8* male mice and double heterozygous *Rnf8;Scml2* female mice were bred to produce *Rnf8;Scml2*-double knockout (dKO) male pups. All animals were handled in strict accordance with good animal practice as defined by the relevant national animal welfare bodies. All experimental work was approved by the Institutional Animal Care and Use Committee protocol no. IACUC2015-0032.

### Size measurement and analysis of testes

To evaluate the sizes of testes between mouse models, the weight of the 2 testes obtained from a mouse were recorded in milligrams and summed; then, this value was divided by the mouse body weight recorded in grams. Data were collected from 9, 5, 7, and 7 independent mice from each mouse model (wild-type, *Rnf8*-KO, *Scml2*-KO, and *Rnf8*;*Scml2*-KO, respectively) for comparison at the ages of 6–48 weeks postpartum. Statistical analyses were performed using Prism 7 (GraphPad); data underwent unpaired t-test between each mouse line.

### Histology, immunohistochemistry, hematoxylin and eosin staining, and imaging

For preparation of testicular paraffin blocks, testes of mutants and littermate controls were fixed with 4% paraformaldehyde at 4°C overnight. Testes were then dehydrated and embedded in paraffin. For histological analyses, 6 μm-thick paraffin sections were deparaffinized and autoclaved in Target Retrieval Solution (DAKO; S-1700) at 121°C for 20 min. The sections were blocked with Blocking One Histo (Nacalai USA; 06349–64) for 10 min at room temperature, and then incubated with primary antibodies at 4°C overnight. The following primary antibodies were used at the described dilutions: rabbit monoclonal anti-Wilm’s Tumor 1, WT1 (Abcam; ab89901), 1:200; mouse monoclonal anti-promyelocytic leukemia zinc finger antibody, PLZF (Santa Cruz Biotechnology; sc-28319), 1:100; guinea pig polyclonal anti-testis-specific histone H1, H1T (gift from Mary Ann Handel), 1:500; mouse monoclonal anti-phosphorylated H2AX (Ser139), γH2AX (Millipore; 05–636), 1:2500; rabbit polyclonal anti-acetylated-histone H3 (Lys27), H3K27ac (Diagenode; C15410196), 1:50; and rabbit polyclonal anti-dimethyl-Histone H3 (Lys4), H3K4me2 (Millipore; 07–030), 1:100. Resulting signals were detected by incubation with secondary antibodies: Alexa Fluors 488 (ThermoFisher Scientific; A-11017 or A-11070) and 594 (ThermoFisher Scientific; A-11020 or A-11072). Sections were counterstained with DAPI (1 μg/ml). Images were obtained with a Nikon Eclipse Ti-E microscope equipped with a Zyla 5.5 sCMOS camera (Andor Technology) using a 20x Plan Apo objective, NA 0.75 (Nikon). Images were processed using NIS-Elements (Nikon) and Photoshop (Adobe) software. For analysis, a minimum of 10 images per experimental group was captured. For hematoxylin and eosin (H&E) staining, slides were deparaffinized and then placed in hematoxylin for 10 min. Then, slides were rinsed in warm water for 10 min and placed in eosin stain for 10 min. Following dehydration, coverslips were mounted on slides with mounting medium (Fisher Scientific; SP15-500). All images of H&E-stained sections were acquired with a Nikon Eclipse E800 microscope equipped with a Nikon DXM1200 digital camera using a 20x Plan Fluor objective, NA 0.50 (Nikon). Image acquisition was performed using NIS-Elements (Nikon) software. For analysis, a minimum of 5 images per experimental group was captured.

### Meiotic chromosome spreads, staging of meiotic prophase, immunocytochemistry, and imaging

Meiotic chromosome spreads were prepared as previously described [40, 47]. For immunostaining experiments, surface spreads were washed in PBST for 30 min at room temperature and blocked with antibody dilution buffer (0.15% BSA, 0.1% Tween 20 in PBS) for 30 min at room temperature. Primary antibodies were added to surface spreads and incubated overnight in humid chambers at room temperature. The following primary antibodies were used with the corresponding dilutions: mouse monoclonal anti-SYCP3 (Abcam; ab97672), 1:5000; rabbit polyclonal anti-SYCP3 (Novus; NB300-231), 1:500; rabbit polyclonal anti-SYCP1 (Abcam, ab15090), 1:1500; mouse monoclonal anti-ubiquitinated proteins, clone FK2 (Millipore; 04–263), 1:500; rabbit polyclonal anti-monoubiquitinated histone H2A, H2AK119ub (clone D27C4; Cell Signaling Technology; #8240), 1:1000; mouse monoclonal anti-monoubiquitinated histone H2A, clone E6C5 (Millipore; 05–678), 1:1000; rabbit polyclonal anti-acetylated histone H3 Lys27, H3K27ac (Active Motif; 39133), 1:500; and rabbit polyclonal anti-dimethyl histone H3 Lys4, H3K4me2 (Millipore; 07–030), 1:500. The slides were incubated for 1 h at room temperature in humid chambers in darkness with various combinations of the following secondary antibodies: Alexa Fluors 488, 594, 647 (ThermoFisher Scientific; A-21237, A-21246; Jackson ImmunoResearch; 706-606-148), and/or Cy3 (Jackson ImmunoResearch; 115-167-003 or 111-166-003). Slides were mounted with #1.5 thickness coverslips (ThermoFisher Scientific; 12-544G) using ProLong Gold (ThermoFisher Scientific; P36930) after incubation in PBST containing DAPI (1 μg/ml) for 10 min at room temperature in darkness. Images were obtained with a Nikon Eclipse Ti-E microscope equipped with a Zyla 5.5 sCMOS camera (Andor Technology) and a 60x CFI Apochromat TIRF oil immersion objective, NA 1.4 (Nikon). Images were processed with NIS-Elements (Nikon), Fiji ImageJ (NIH [48]), Photoshop (Adobe), and Illustrator (Adobe) software. A minimum of 30 spermatocyte nuclei images per substage of meiotic prophase, from at least 3 independent mice per mouse model, was captured for analysis.

Analysis of chromosome spreads included comparison of accumulation, depletion, and relative intensity patterns on the sex chromosomes using antibodies of interest, and between matched substages of meiotic prophase between the four mouse models. Stages of spreads were distinguished by immunostaining with anti-SYCP3 antibody as previously described [40]. To dissect the dynamic accumulation of different factors on the sex chromosomes in late prophase, we categorized diplotene spermatocytes into two stages: early and late. Briefly, early diplotene spermatocytes were distinguished by partial desynapsis of <50% of autosomes and the stretched status of sex chromosome axes; late diplotene spermatocytes were distinguished by increasingly broad desynapsis of >50% of autosomes and the compaction of sex chromosome axes. To generate the line traces, we exported the adjusted images to the NIH’s ImageJ software and performed the quantitative analysis along a single transect as shown as performed previously [27]. Round spermatids were examined with slides preserving the relative nuclear organization of spermatogenic cells, prepared as previously described [20, 49, 50] and imaged as described above except with a 100x CFI Apochromat TIRF oil immersion objective, NA 1.4 (Nikon).

### Germ cell fractionation

For ChIP-seq, pachytene spermatocytes and round spermatids were isolated from wild-type testes through BSA gravity sedimentation as described [51]. To perform RNA-seq of cells isolated from *Rnf8;Scml2-*dKO testes, the same method of BSA gravity sedimentation was performed on a small scale, which enabled the purification of PS and RS from a single male mouse. Briefly, a pair of testes from one *Rnf8;Scml2-*dKO mouse underwent digestion by treatments with collagenase and trypsin, along with DNase I. The cells were isolated and suspended in Krebs-Ringer Bicarbonate Buffer containing 0.5% BSA. Subsequently, the cell suspension was loaded into a gradient of Krebs-Ringer Bicarbonate Buffer containing 2% and 4% BSA, generated by a gradient maker (VWR; GM-100). The cell suspension was allowed to settle for 3 h at 4°C before fractions were collected. Purity was confirmed by nuclear staining of a sample aliquot of each collected fraction with Hoechst 33342 via fluorescence microscopy. Greater than 90% purity was confirmed for each purification.

### ChIP-seq and data analysis

Cells were suspended in chilled 1x PBS. One-eleventh volume of crosslinking solution (50 mM HEPES-NaOH pH 7.9, 100 mM NaCl, 1 mM EDTA, 0.5 mM EGTA, and 8.8% formaldehyde) was added to the cell suspension and incubated on ice for 8 min. One-twentieth volume of 2 M glycine was added to the cell suspension and incubated at room temperature for 5 min to stop the reaction. Cells were washed twice with PBS, frozen at -80°C, and lysed at 4°C for 10 min each in ChIP lysis buffer 1 (50 mM HEPES pH 7.9, 140 mM NaCl, 10% glycerol, 0.5% IGEPAL-630, 0.25% Triton X-100). After centrifugation at 2,000x*g* for 10 min at 4°C, pellets were resuspended with ChIP lysis buffer 2 (10 mM Tris-HCl pH 8.0, 200 mM NaCl, 1 mM EDTA, 0.5 mM EGTA) and incubated at 4°C for 10 min. After centrifugation at 2,000x*g* for 10 min at 4°C, pellets were washed with TE containing 0.1% SDS and protease inhibitors (Sigma; 11836145001), and resuspended with the same buffer. Chromatin was sheared to approximately 200–500 bp by sonication using a Covaris sonicator at 10% duty cycle, 105 pulse intensity, 200 burst for 2 min. Sheared chromatin was cleared by centrifugation at 20,000x*g* for 20 min, followed by pre-incubation with Dynabeads Protein G. Chromatin immunoprecipitation was carried out on an SX-8X IP-STAR compact automated system (Diagenode). Briefly, Dynabeads Protein G were pre-incubated with 0.1% BSA for 2 h. Then, the cleared chromatin was incubated with beads conjugated to antibodies against H3K27ac (Active Motif; 39133) at 4°C for 8 h, washed sequentially with wash buffer 1 (50 mM Tris-HCl pH 8.0, 150 mM NaCl, 1 mM EDTA, 0.1% SDS, 0.1% NaDOC, and 1% Triton X-100), wash buffer 2 (50 mM Tris-HCl pH 8.0, 250 mM NaCl, 1 mM EDTA, 0.1% SDS, 0.1% NaDOC, and 1% Triton X-100), wash buffer 3 (10 mM Tris-HCl pH 8.0, 250 mM LiCl, 1 mM EDTA, 0.5% NaDOC, and 0.5% NP-40), wash buffer 4 (10 mM Tris-HCl pH 8.0, 1 mM EDTA, and 0.2% Triton X-100), and wash buffer 5 (10 mM Tris-HCl). DNA libraries were prepared through the ChIPmentation method [52]. Briefly, beads were resuspended in 30 μl of the tagmentation reaction buffer (10 mM Tris-HCl pH 8.0 and 5 mM MgCl_2_) containing 1 μl Tagment DNA Enzyme from the Nextera DNA Sample Prep Kit (Illumina) and incubated at 37°C for 10 min in a thermal cycler. The beads were washed twice with 150 μl cold wash buffer 1, incubated with elution buffer (10 mM Tris-HCl pH 8.0, 1 mM EDTA, 250 mM NaCl, 0.3% SDS, 0.1 μg/μl Proteinase K) at 42°C for 30 min, and then incubated at 65°C for another 5 h to reverse cross-linking. DNA was purified with the MinElute Reaction Cleanup Kit (Qiagen) and amplified with NEBNext High-Fidelity 2x PCR Master Mix (NEB). Amplified DNA was purified by Agencourt AMPure XP (Beckman Coulter). Afterward, DNA fragments in the 250- to 500-bp size range were prepared by agarose gel size selection. DNA libraries were adjusted to 5 nM in 10 mM Tris-HCl pH 8.0 and sequenced with an Illumina HiSeq 2500.

Data analysis was performed in the Wardrobe Experiment Management System (https://code.google.com/p/genome-tools/ [53]). For the analysis of H3K4me2, we used our publish data [42]. Briefly, reads were aligned to the mouse genome (mm10) with Bowtie (version 1.0.0 [54]) and displayed on a local mirror of UCSC genome browser as coverage. Islands of H3K27ac- and H3K4me2-enrichment were identified using MACS2 (version 2.0.10.20130712 [55]). MAnorm, software designed for the quantitative comparison of ChIP-seq datasets [43], was applied to compare the enrichment profile of H3K27ac or H3K4me2 between pachytene spermatocytes and round spermatids.

### RNA-seq

Total RNA was purified from pachytene spermatocytes or round spermatids using an RNeasy Micro Kit (Qiagen) according to the manual provided. RNA quality and quantity were checked via Bioanalyzer (Agilent) and Qubit (Life Technologies), respectively. The initial amplification step was performed with the Ovation RNA-Seq System v2 (NuGEN). The assay was used to amplify RNA samples and create double-stranded cDNA. Libraries were then created with the Nextera XT DNA Sample Preparation Kit (Illumina) and sequenced with an Illumina HiSeq 2500.

### RNA-seq data analysis

The RNA-seq results of *Rnf8;Scml2-*dKO cells (two-biological replicates) were analyzed with our previous data obtained from wild-type, *Rnf8-*KO, and *Scml2-*KO mice (GSE55060, GSE69946) [35, 42]. Data analysis for RNA-seq was performed in the Wardrobe Experiment Management System. [56]. FASTQ files from the Illumina pipeline were aligned via the Spliced Transcripts Alignment to a Reference (STAR) software (version STAR_2.4.2a) [57] with the following parameters:--outFilterMultimapNmax 1--outFilterMismatchNmax 2 (to see the full manual, go to this STAR GitHub page: https://github.com/alexdobin/STAR/blob/master/doc/STARmanual.pdf?raw=true). RefSeq annotation from the UCSC genome browser (11/2012) [58] for the mm10 genome was used. The--outFilterMultimapNmax parameter was used to allow unique alignment only, and the--outFilterMismatchNmax parameter was used to allow a maximum of only 2 errors. All reads from resulting bam files were split for related isoforms with respect to RefSeq annotation. Then, an expectation-maximization algorithm was used to estimate appropriate numbers of reads for each isoform [56]. To estimate differences between experiments, the DESeq2 package [59] was used.

### Data access

ChIP-seq and RNA-seq datasets generated in this study were deposited to the NCBI Gene Expression Omnibus (GEO; http://www.ncbi/nlm.nih.gov/geo/) under accession number GSE107398.

## Supporting information

S1 Table RNA-seq analysis of pachytene spermatocytes from wild-type, *Rnf8*-KO, *Scml2*-KO, and *Rnf8*;*Scml2*-KO testes. (XLSX)

S2 Table RNA-seq analysis of round spermatids from wild-type, *Rnf8*-KO, *Scml2*-KO, and *Rnf8*;*Scml2*-KO testes. (DOCX)

S3 Table Gene lists of down-regulated genes in round spermatids of *Rnf8*-KO, *Scml2*-KO, and *Rnf8*;*Scml2*-KO. (XLSX)

S1 Fig Spermatogenic phenotypes of *Rnf8;Scml2*-dKO mice. (**A**) Ratio of total testicular weight: total testes weight (sum of 2 testes per mouse model; mg)/body weight (g) of each mouse model. Data were collected from 9, 5, 7, and 7 independent mice from each mouse model (wild-type, *Rnf8*-KO, *Scml2*-KO, and *Rnf8*;*Scml2*-KO, respectively) for comparison at the ages of 6–48 weeks postpartum. ** *P* < 0.01, *** *P* < 0.001, **** *P* < 0.0001, Unpaired t-test. Bars represent S.D. (**B**) Fertility test. Four *Rnf8;Scml2*-dKO male mice were paired with one wild-type C57Bl/6 female mouse each for a breeding period of 90 days. The four *Rnf8;Scml2*-dKO breeding pairs generated no litters; on the other hand, the littermate control breeding pairs (wild-type: n = 2, and *Rnf8*+/-: n = 2) were fertile. Bars represent S.E.M. Unpaired t test. (**C**) Immunostaining of chromosome spreads with antibodies against SYCP3 and SYCP1, a factor present at synapsed meiotic axes. Ectopic asynapsis was not significantly increased in the *Rnf8;Scml2*-dKO. Three independent littermate pairs were analyzed. n.s.: not significant, Fisher’s exact test. (**D**) Testicular sections stained with hematoxylin and eosin (H&E) staining. (**E**, **F**) Immunostaining of testicular sections with WT1, a marker of Sertoli cells, and PLZF, a marker of undifferentiated spermatogonia (**E**); and immunostaining with H1T, a germ cell marker that detects germ cells after the mid pachytene stage, and γH2AX, a maker of meiosis that detects spermatocytes in the leptotene and zygotene stages, and sex chromosomes in the pachytene and diplotene stages (**F**). These results indicate more profound testicular defects than that of single mutants for *Rnf8* and *Scml2*, although undifferentiated spermatogonia and late germ cells remained in the double mutants. DNA was counterstained with DAPI (1 μg/ml). Scale bar: 100 μm. (**G**) Frequency of tubules with H1t-positive spermatids (%). Two independent mice were analyzed. n.s.: not significant, Fisher’s exact test.
(TIF)

S2 Fig Additional example of immunostaining with D27C4 antibody, which recognizes H2AK119ub. Immunostaining of SYCP3 and D27C4 (H2AK119ub) on meiotic chromosome spreads. The areas surrounding sex chromosomes are shown in dotted boxes. Scale bar: 10 μm. The intensity of immunostaining is quantified by densitometry across the indicated path (‘ to”) and plotted in a relative intensity range of 0–1, which is normalized among all images in this figure, Fig 2 and Fig 3. DAPI-stained XY bodies were observed, while H2AK119ub were largely decreased on XY bodies in wild-type and *Rnf8*-KO mice.
(TIF)

S3 Fig The DAPI-discernible structure of PMSC was not disrupted in *Rnf8;Scml2*-dKO round spermatids. (**A, B**) Immunostaining of testicular paraffin sections with anti-H3K4me2 and anti-H3K27ac antibodies. Slides were counterstained with DAPI. Regions bordered by dashed squares are magnified in the right panels. Arrowheads: PMSC. Bars in left panels: 50 μm; bars in right panels: 5 μm.
(TIF)

S4 Fig Reproducibility of H3K27ac ChIP-seq and MAnorm analysis of ChIP-seq peaks. (**A**) Two-dimensional scatter plots showing the reproducibility of H3K27ac ChIP-seq signals at individual peaks between biological replicates. Each peak was identified using MACS (P < 1×10^−5^). Enrichment levels for H3K27ac ChIP-seq are shown in Log2 RPKM values. The color scale indicates the density of H3K27ac ChIP-seq. Pearson correlation values are shown. (**B**) MAnorm analysis of ChIP-seq peaks between H3K4me2 and H3K27ac in PS and RS of wild-type mice. The genomic distribution of each peak is shown with colored bars.
(TIF)
