# Supplementary material for: RNF8 and SCML2 cooperate to regulate ubiquitination and H3K27 acetylation for escape gene activation on the sex chromosomes
Source: PLoS Genet. 2018 Feb 20;14(2):e1007233. doi: 10.1371/journal.pgen.1007233 (PMC5834201; doi:10.1371/journal.pgen.1007233)
Supplement: S2 Table — (DOCX) [file pgen.1007233.s002.docx]

**List of *Ae. aegypti* miRNAs that have significant potential to bind to the 5’ or 3’ UTR of the DENV genome**

AF038403.3prime aae-bantam-3p.fasta

MFE of real data: -20.5

MFEs of simulated data range from -24.9 to -10.2

Real MFE is better than 96.3% of simulated MFEs *****

--

AF038403.5prime aae-mir-1.fasta

MFE of real data: -20.5

MFEs of simulated data range from -26.1 to -13.0

Real MFE is better than 95.9% of simulated MFEs *****

--

AF038403.3prime aae-mir-10.fasta

MFE of real data: -22.1

MFEs of simulated data range from -29.5 to -10.2

Real MFE is better than 95.5% of simulated MFEs *****

--

AF038403.3prime aae-mir-263a-5p.fasta

MFE of real data: -22.5

MFEs of simulated data range from -28.3 to -10.5

Real MFE is better than 95.7% of simulated MFEs *****

--

AF038403.3prime aae-mir-281-5p.fasta

MFE of real data: -21.4

MFEs of simulated data range from -25.7 to -11.7

Real MFE is better than 96.5% of simulated MFEs *****

--

AF038403.5prime aae-mir-282-5p.fasta

MFE of real data: -32.7

MFEs of simulated data range from -41.4 to -22.4

Real MFE is better than 95.4% of simulated MFEs *****

--

AF038403.3prime aae-mir-282-5p.fasta

MFE of real data: -27.8

MFEs of simulated data range from -38.6 to -14.4

Real MFE is better than 96.5% of simulated MFEs *****

--

AF038403.5prime aae-mir-285.fasta

MFE of real data: -23.3

MFEs of simulated data range from -28.6 to -13.4

Real MFE is better than 97.5% of simulated MFEs *****

--

AF038403.5prime aae-mir-2945-5p.fasta

MFE of real data: -31.4

MFEs of simulated data range from -36.5 to -21.3

Real MFE is better than 96.5% of simulated MFEs *****

--

AF038403.3prime aae-mir-2a-5p.fasta

MFE of real data: -20.8

MFEs of simulated data range from -26.5 to -11.8

Real MFE is better than 95.1% of simulated MFEs *****

--

AF038403.5prime aae-mir-308-3p.fasta

MFE of real data: -22.5

MFEs of simulated data range from -24.3 to -12.3

Real MFE is better than 99.6% of simulated MFEs *****

--

AF038403.5prime aae-mir-8-5p.fasta

MFE of real data: -27.9

MFEs of simulated data range from -33.3 to -16.3

Real MFE is better than 99.6% of simulated MFEs *****

--

AF038403.5prime aae-mir-965.fasta

MFE of real data: -29.9

MFEs of simulated data range from -29.8 to -16.4

Real MFE is better than 100% of simulated MFEs *****

--

AF038403.3prime aae-mir-965.fasta

MFE of real data: -23.3

MFEs of simulated data range from -26.7 to -11.4

Real MFE is better than 98.3% of simulated MFEs *****

--

AF038403.5prime aae-mir-998.fasta

MFE of real data: -22.7

MFEs of simulated data range from -28.1 to -13.4

Real MFE is better than 97.4% of simulated MFEs *****

--

AF038403.3prime aae-mir-999.fasta

MFE of real data: -23.2

MFEs of simulated data range from -27.6 to -11.3

Real MFE is better than 99.2% of simulated MFEs *****

--

AF038403.5prime aae-mir-9a.fasta

MFE of real data: -26.8

MFEs of simulated data range from -33.3 to -17.4

Real MFE is better than 97.4% of simulated MFEs *****

--

AF038403.5prime aae-mir-9b.fasta

MFE of real data: -26.7

MFEs of simulated data range from -30.9 to -18.1

Real MFE is better than 96.3% of simulated MFEs *****
