# Supplementary figures and images for: RNF8 and SCML2 cooperate to regulate ubiquitination and H3K27 acetylation for escape gene activation on the sex chromosomes
Source: PLoS Genet. 2018 Feb 20;14(2):e1007233. doi: 10.1371/journal.pgen.1007233 (PMC5834201; doi:10.1371/journal.pgen.1007233)

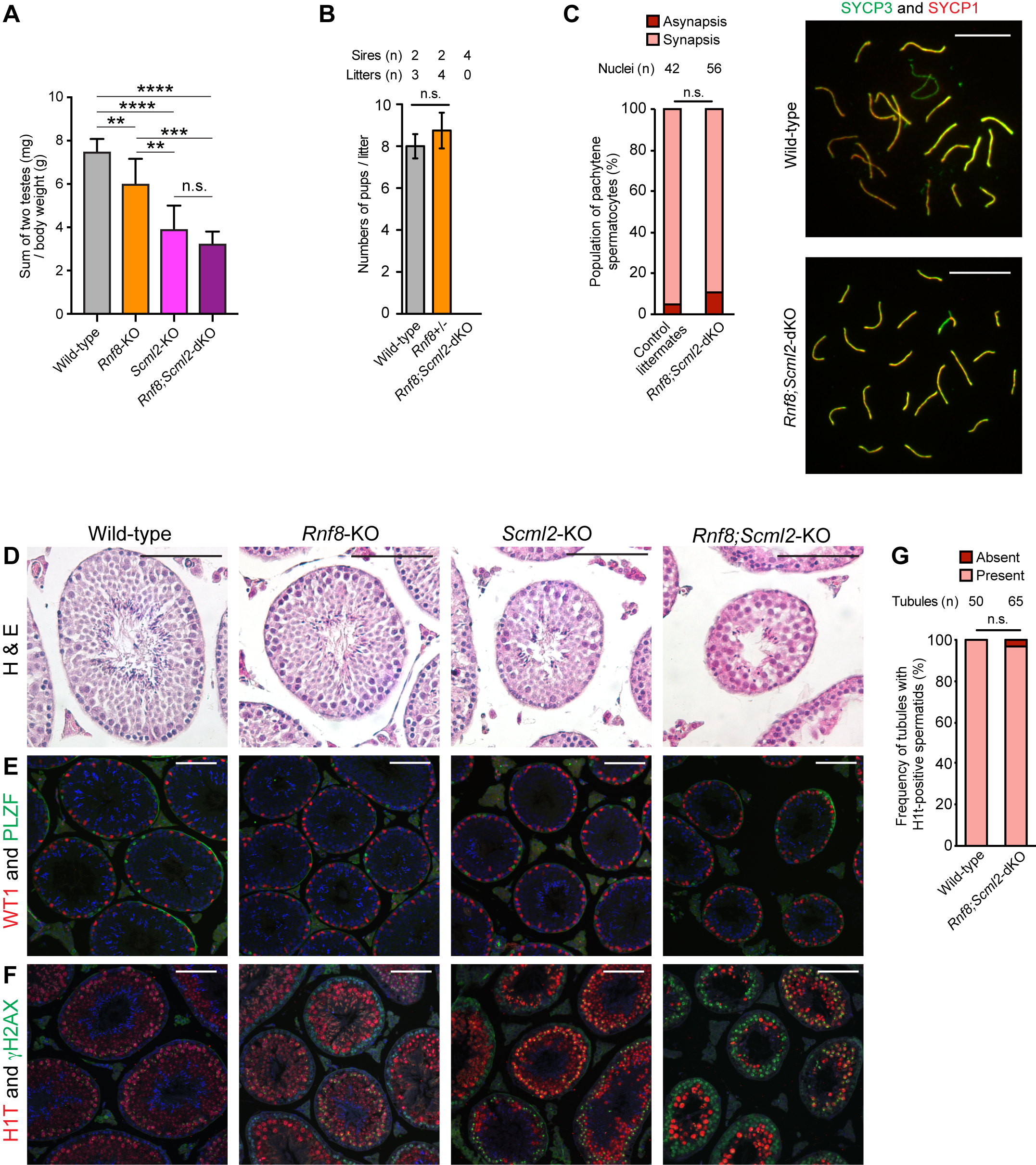

Supplement: S1 Fig — (A) Ratio of total testicular weight: total testes weight (sum of 2 testes per mouse model; mg)/body weight (g) of each mouse model. Data were collected from 9, 5, 7, and 7 independent mice from each mouse model (wild-type, Rnf8-KO, Scml2-KO, and Rnf8;Scml2-KO, respectively) for comparison at the ages of 6–48 weeks postpartum. ** P < 0.01, *** P < 0.001, **** P < 0.0001, Unpaired t-test. Bars represent S.D. (B) Fertility test. Four Rnf8;Scml2-dKO male mice were paired with one wild-type C57Bl/6 female mouse each for a breeding period of 90 days. The four Rnf8;Scml2-dKO breeding pairs generated no litters; on the other hand, the littermate control breeding pairs (wild-type: n = 2, and Rnf8+/-: n = 2) were fertile. Bars represent S.E.M. Unpaired t test. (C) Immunostaining of chromosome spreads with antibodies against SYCP3 and SYCP1, a factor present at synapsed meiotic axes. Ectopic asynapsis was not significantly increased in the Rnf8;Scml2-dKO. Three independent littermate pairs were analyzed. n.s.: not significant, Fisher’s exact test. (D) Testicular sections stained with hematoxylin and eosin (H&E) staining. (E, F) Immunostaining of testicular sections with WT1, a marker of Sertoli cells, and PLZF, a marker of undifferentiated spermatogonia (E); and immunostaining with H1T, a germ cell marker that detects germ cells after the mid pachytene stage, and γH2AX, a maker of meiosis that detects spermatocytes in the leptotene and zygotene stages, and sex chromosomes in the pachytene and diplotene stages (F). These results indicate more profound testicular defects than that of single mutants for Rnf8 and Scml2, although undifferentiated spermatogonia and late germ cells remained in the double mutants. DNA was counterstained with DAPI (1 μg/ml). Scale bar: 100 μm. (G) Frequency of tubules with H1t-positive spermatids (%). Two independent mice were analyzed. n.s.: not significant, Fisher’s exact test. (TIF) [file pgen.1007233.s004.tif]

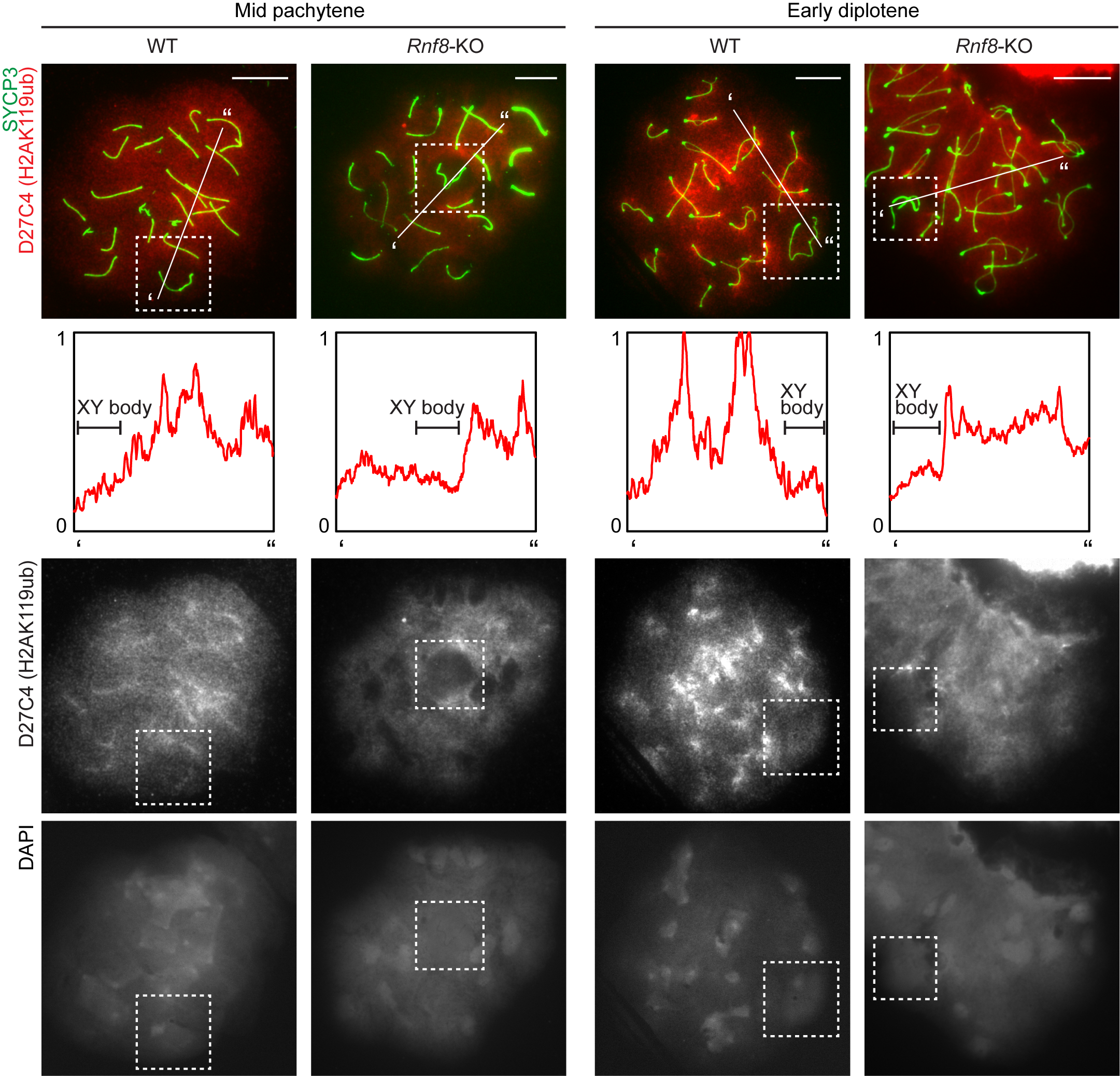

Supplement: S2 Fig — Immunostaining of SYCP3 and D27C4 (H2AK119ub) on meiotic chromosome spreads. The areas surrounding sex chromosomes are shown in dotted boxes. Scale bar: 10 μm. The intensity of immunostaining is quantified by densitometry across the indicated path (‘ to”) and plotted in a relative intensity range of 0–1, which is normalized among all images in this figure, Fig 2 and Fig 3. DAPI-stained XY bodies were observed, while H2AK119ub were largely decreased on XY bodies in wild-type and Rnf8-KO mice. (TIF) [file pgen.1007233.s005.tif]

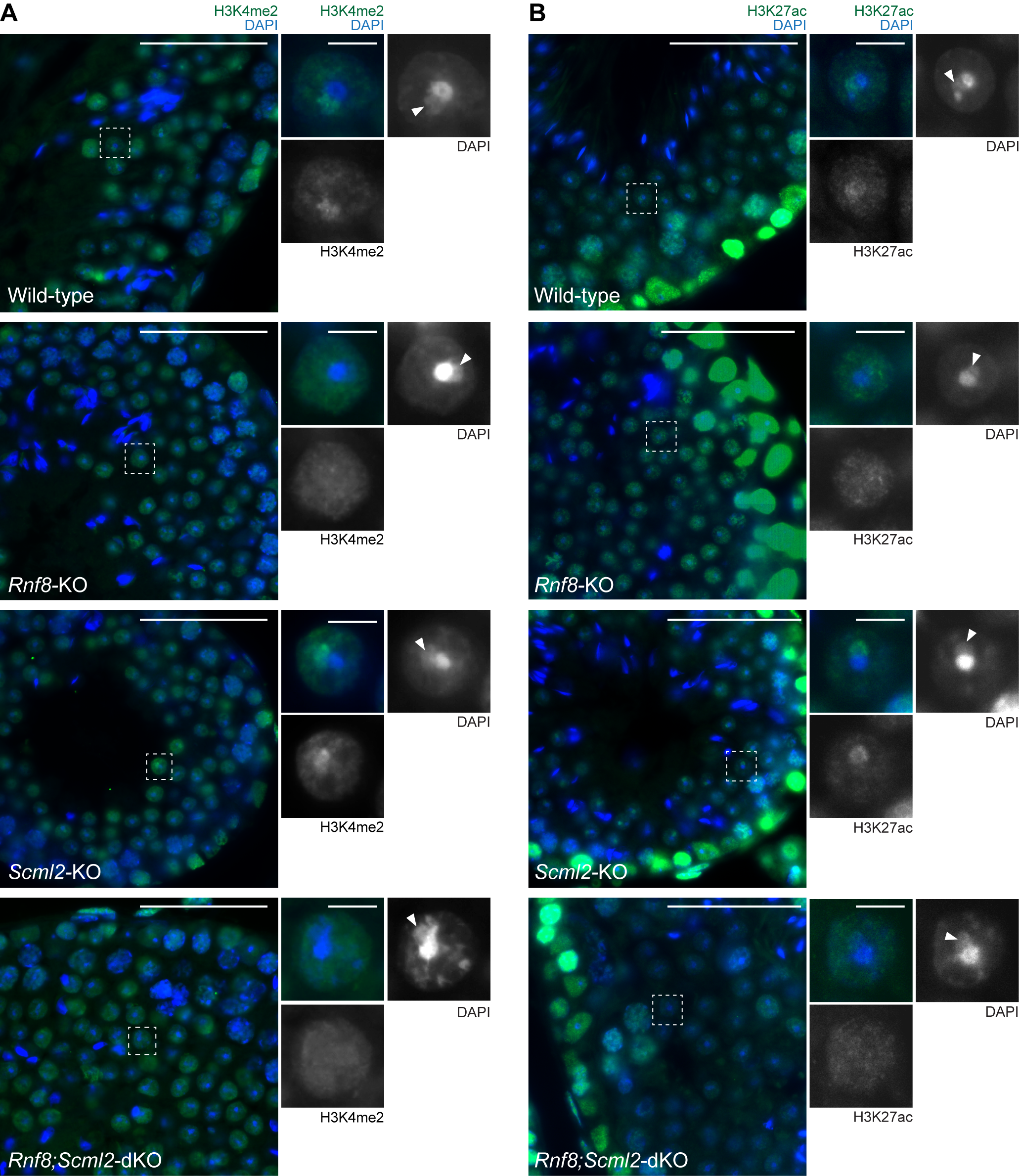

Supplement: S3 Fig — (A, B) Immunostaining of testicular paraffin sections with anti-H3K4me2 and anti-H3K27ac antibodies. Slides were counterstained with DAPI. Regions bordered by dashed squares are magnified in the right panels. Arrowheads: PMSC. Bars in left panels: 50 μm; bars in right panels: 5 μm. (TIF) [file pgen.1007233.s006.tif]

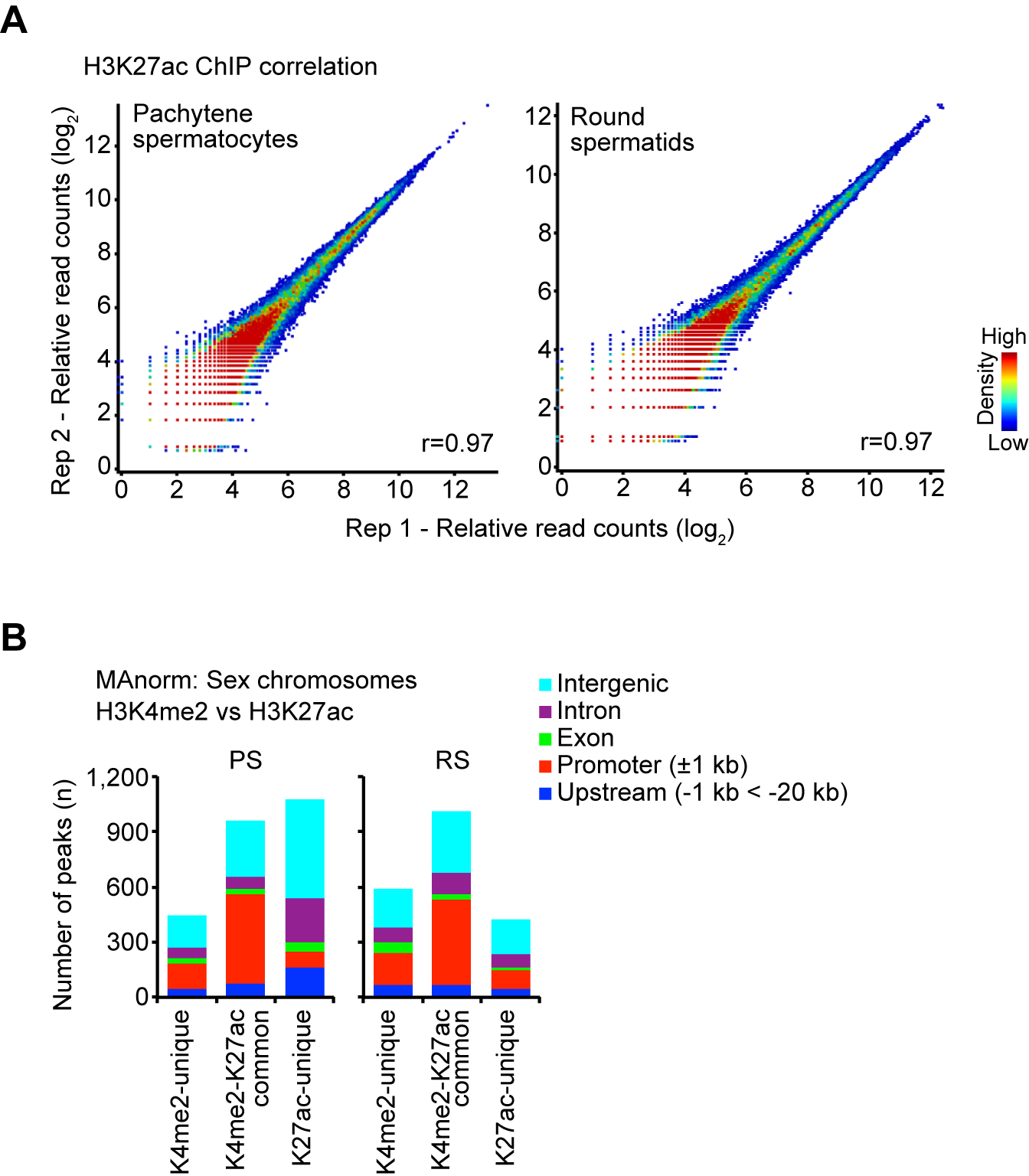

Supplement: S4 Fig — (A) Two-dimensional scatter plots showing the reproducibility of H3K27ac ChIP-seq signals at individual peaks between biological replicates. Each peak was identified using MACS (P < 1×10^−5^). Enrichment levels for H3K27ac ChIP-seq are shown in Log2 RPKM values. The color scale indicates the density of H3K27ac ChIP-seq. Pearson correlation values are shown. (B) MAnorm analysis of ChIP-seq peaks between H3K4me2 and H3K27ac in PS and RS of wild-type mice. The genomic distribution of each peak is shown with colored bars. (TIF) [file pgen.1007233.s007.tif]
